# The Expanded Endocannabinoid System Contributes to Metabolic and Body Mass Shifts in First-Episode Schizophrenia: A 5-Year Follow-Up Study

**DOI:** 10.3390/biomedicines10020243

**Published:** 2022-01-24

**Authors:** Madis Parksepp, Liina Haring, Kalle Kilk, Kadri Koch, Kärt Uppin, Raul Kangro, Mihkel Zilmer, Eero Vasar

**Affiliations:** 1 Institute of Clinical Medicine, University of Tartu, 50406 Tartu, Estonia; Madis.Parksepp@vmh.ee; 2 Psychiatry Clinic of Viljandi Hospital, 71024 Viljandi, Estonia; 3 Psychiatry Clinic of Tartu University Hospital, 50406 Tartu, Estonia; Kadri.Koch@kliinikum.ee (K.K.); Kart.Uppin@kliinikum.ee (K.U.); 4 Centre of Excellence for Genomics and Translational Medicine, Institute of Biomedicine and Translational Medicine, Univesignallingrsity of Tartu, 50090 Tartu, Estonia; Kalle.Kilk@ut.ee (K.K.); Mihkel.Zilmer@ut.ee (M.Z.); Eero.Vasar@ut.ee (E.V.); 5 Institute of Mathematics and Statistics, University of Tartu, 50090 Tartu, Estonia; Raul.Kangro@ut.ee

**Keywords:** endocannabinoid system, endocannabinoids, *N*-acylethanolamines, phosphatidylcholines, CB1 receptors, PPARs, antipsychotic treatment, first-episode psychosis, schizophrenia spectrum disorders

## Abstract

Alterations in the expanded endocannabinoid system (eECS) and cell membrane composition have been implicated in the pathophysiology of schizophrenia spectrum disorders. We enrolled 54 antipsychotic (AP)-naïve first-episode psychosis (FEP) patients and 58 controls and applied a targeted metabolomics approach followed by multivariate data analysis to investigate the profile changes in the serum levels of endocannabinoids: 2-arachidonoylglycerol (2-AG) and anandamide, endocannabinoids-like *N*-acylethanolamines (NAEs: linoleoylethanolamide, oleoylethanolamide, and palmitoylethanolamide), and their dominating lipid precursor’s phosphatidylcholines. Biomolecule profiles were measured at the onset of first-episode psychosis (FEP) and 0.6 years and 5.1 years after the initiation of AP treatment. The results indicated that FEP might be characterized by elevated concentrations of NAEs and by decreased 2-AG levels. At this stage of the disease, the NAE-mediated upregulation of peroxisome proliferator-activated receptors (PPARs) manifested themselves in energy expenditure. A 5-year disease progression and AP treatment adverse effects led to a robust increase in 2-AG levels, which contributed to strengthened cannabinoid (CB1) receptor-mediated effects, which manifested in obesity. Dynamic 2-AG, NAEs, and their precursors in terms of phosphatidylcholines are relevant to the description of the metabolic shifts resulting from the altered eECS function during and after FEP.

## 1. Introduction

Schizophrenia spectrum disorders (SSD) are chronic psychiatric conditions consisting of a diversity of clinical features [1]. Despite ongoing progress, the pathophysiology of SSD remains highly debated, and so does its treatment, with most patients experiencing long term disability, severe morbidity, and increased mortality rates compared to the general population [2]. One of the reasons for the shortened life expectancy is the continuous treatment of patients with antipsychotics (APs) associated with excessive weight gain and other metabolic abnormalities, such as hyperglycaemia, hyperinsulinemia, dyslipidaemia, and metabolic syndrome [3]. However, unraveling the pathophysiology of SSD is challenging, as psychotic syndromes are heterogeneous. Several theoretical frameworks have been proposed to explain the pathological basis of the SSD; among others, the phospholipid hypothesis and cannabinoid hypothesis [4,5,6]. The first of them suggests that a deficient uptake or excessive breakdown of membrane phospholipids (PLs) or changes in the membrane PL composition may be a biochemical basis for the neurodevelopmental concept of SSD [4]. The mechanisms that regulate the metabolism of highly unsaturated fatty acids (FAs) at the Sn2 position of PL molecules result in abnormalities in the structure of the neuronal membrane and cellular signaling, and, thereby, cause secondary disturbances in the mechanisms involved in neurotransmission [7]. The second, the cannabinoid theory, emphasizes the importance of an endogenous and exogenous perspective in the development of the disorder, according to which, the dysregulation of the endocannabinoid system (ECS) may contribute to the pathophysiology of SSD, and the risk associated with cannabis abuse could facilitate the onset of the disorder in vulnerable individuals or aggravate the symptoms in SSD patients [6,8]. In addition, ECS plays a very important role in the systemic and cellular regulation of metabolism and may, thereby, affect the onset and persistence of metabolic shifts that are often associated with AP therapy. Furthermore, the ECS is one of the most widely distributed neurotransmitter systems in the brain, with a critical neuromodulatory role [9], as it is intertwined with other lipid or non-lipid signaling pathways and associated with other regulatory networks [10]. The classical ECS comprises two inhibitory G-protein-coupled receptors (GPRs), cannabinoid receptors type 1 and 2 (CB1, CB2), and two major endocannabinoid (eCBs) ligands for CB1 and CB2 known as 2-arachidonoylglycerol (2-AG) and *N*-arachidonoylethanolamine (anandamide, AEA). These derivates of arachidonic acid (C20:4) are the most studied eCBs [11].

Expanded ECS (eECS) comprises several bioactive metabolites, including endocannabinoids-like (eCB-like) *N*-acylethanolamines (NAEs), among other polyunsaturated NAE (linoleoylethanolamide- LEA, C18:2), and monounsaturated and saturated NAEs (oleoylethanolamide- OEA, C18:1 and palmitoylethanolamide- PEA, C16:0, respectively). These signaling lipid-related compounds share the biosynthetic and degradative pathways and target G-protein receptors (e.g., GPR119 and GPR55), transient receptor potential vanilloid 1 (TRPV1), and nuclear peroxisome proliferator-activated receptors (PPARs), but not CB1 and CB2 [12]. The PPARs family includes PPAR-α, PPAR-γ, and PPAR-β/δ [13]. The individual PPARs interact with each other and demonstrate distinct actions, regulating the expression of several target genes involved in energy homeostasis, lipid uptake and metabolism, insulin sensitivity, other metabolic functions, immune reaction, cell differentiation, and a variety of other cellular changes and adaptive responses [14,15]. PPARs are activated by FAs (unsaturated, monounsaturated, and PUFAs), eicosanoids, sphingolipids, and NAEs [16].

The biosynthesis of eCBs and eCB-like NAEs have many steps initiated by the synthesis of glycero-PLs from the dietary FA pool [17]. These biomolecules act as retrograde messengers that inhibit the neurotransmitter release at both inhibitory and excitatory synapses, thereby mediating various forms of long- and short-term plasticity in the brain [18,19].

The correct interaction between all of these eECS elements plays an important role in the central nervous system (CNS) development, synaptic plasticity, and the homeostatic maintenance of many physiological processes, including energy metabolism, immune function, and both central and peripheral nervous system function [20]. This complex signaling system is deeply involved in the onset and progression of pathophysiological conditions, such as systemic inflammation and neuroinflammation [21], obesity and metabolic syndrome [22], and mental disorders, including SSD [23,24,25]. A meta-analysis suggested that the patients at an early stage of illness who were AP-naïve had significantly higher AEA levels in their peripheral blood as compared to the control subjects (CSs) [26]. However, another study demonstrated no changes in the serum AEA and OEA levels in schizophrenia patients [27]. Although the data of serum levels of AEA and OEA can be found in the scientific literature, information about another eCB 2-AG, other eCB-like compounds (e.g., LEA and PEA), and particularly their metabolically relevant lipid-related compounds, such as phosphatidylcholines (PCs), measured simultaneously in SSD patients at different stages of the disease is insufficient.

Based on previous studies, we hypothesized that the eECS may be disrupted in the early stage of SSD. More specifically, we aimed to study the serum concentrations of the main eCBs, eCB-like NAEs, and their metabolically relevant lipid precursors in the first-episode psychosis (FEP) patients compared to CSs, taking advantage of a naturalistic, longitudinal, 5-year follow-up study. Our first aim was to investigate which eCBs and eCB-like NAEs changes occurred at the onset of SSD and after the continuous use of AP treatment to control disease symptoms. The second aim was to identify the profile shifts of targeted lipids (PCs) associated with the changes in eCBs and eCB-like NAEs at different stages of the disease and in the context of AP treatment. To meet the objectives of the study, we simultaneously examined the serum levels of eCBs, eCB-like NAEs, and their lipid precursor profiles differences in drug-naïve patients compared to the CSs; then, to describe the metabolic status changes over the next five years, we investigated the differences in eCBs, eCB-like NAEs, and lipid profiles that occurred in the patients’ group at 0.6 and 5.1 years after initiation of APs.

## 2. Materials and Methods

### 2.1. Participants

A total of 112 adults participated in the study. Patients with FEP (*n* = 54, 59% men) were recruited at the time of their first clinical contact for psychotic symptoms at the Psychiatric Clinic of Tartu University Hospital, Estonia. The inclusion criteria were as follows: patients with FEP, the duration of the untreated psychosis less than 3 years, no AP use before the study, and male or female participants between 18 and 45 years old. If necessary, patients received benzodiazepines the night before the first blood collection at their psychiatrist’s discretion. The exclusions were as follows: patients who had organic or drug-induced psychosis or psychotic disorders due to other medical conditions. FEP diagnoses were based on clinical interviews according to the International Classification of Diseases, Tenth Edition (ICD-10) [28] criteria, and approved by two clinical psychiatrists. Patients’ diagnoses were F23.0 (*n* = 9), F23.1 (*n* = 11), F23.2 (*n* = 15), F23.3 (*n* = 2), F21 (*n* = 1), F20.09 (*n* = 13), and F20.39 (*n* = 3) at baseline. After the recruitment, all FEP patients received AP medication. The history of used APs was collected according to the reviews of patients’ medical charts. No restrictions were made in terms of usage of specific pharmacological substances due to a naturalistic and longitudinal study design. During the study, patients were treated with various doses and types of APs. At the time of the follow-up blood collections, the mean theoretical chlorpromazine equivalent (CPZE) doses according to Gardner et al. [29] were calculated. CPZE is defined as the dose of AP drug, which is equivalent to 100 mg of oral chlorpromazine. Moreover, mood stabilizers, antidepressants, or hypnotics were used according to clinically relevant circumstances.

Patients were examined prospectively. At an average of 0.6-year follow-up, the patient sample consisted of 47 patients (51% men), and at an average of 5.1-year follow-up, the sample comprised 38 patients (43% men). During the monitoring period, the patient dropout rate was 30%. The main reasons for discontinuation were related to their decision to stop AP treatment or because they had changed their place of residence. The patients’ diagnoses at the second follow-up were F20.0 (*n* = 28), F20.1 (*n* = 1), and F25 (*n* = 9).

The control group consisted of 58 subjects who were recruited through advertisements. Of these mentally healthy participants, 44% were male. As it was a naturalistic study, substance abuse was not an exclusion criterion for either group. Twenty-one patients (39%) had smoked cannabis before the FEP. Nineteen patients (35%) reported rare cannabis consumption during the five-year monitoring period, and three of them (men) met the criteria for cannabis use disorder. Fifteen CSs (26%) had tried cannabis at least once during their lifetime. None of them met the criteria of cannabis use disorder. All participants had no history of chronic medical illness and no indications of acute infectious disease at study entry, as evidenced by self-report of symptoms.

Partially, the same FEP and CSs groups were characterized in our previous studies [30,31,32], wherein we focused on describing the characteristics of acylcarnitines and glycerophospholipid profiles in AP-naïve FEPs (*n* = 38) compared to controls (*n* = 37) and demonstrated changes in biomolecular profiles over 0.6 years. We then increased the total number of participants in the study (*n* = 89), collected patient data also at 5 years after initiation of AP therapy, and described changes in amino acid and biogenic amine profiles in the FEP group, before initiating AP therapy over 0.6- and 5-year periods. At present, 5-year longitudinal data from 54 patients and data from 58 controls (partially the same cohort as described previously) were included in the study.

The study was approved by the Ethics Review Committee on Human Research of the University of Tartu, Estonia (initial approval No 177/T-2 and follow-up approval No 211/M-22) and carried out by The Code of Ethics of the World Medical Association. Written informed consent was obtained from all participants.

### 2.2. Procedure

Serum samples and clinical and BMI data of the patients with a psychotic disorder were assessed at three consecutive time points: on admission, at the first follow-up (mean duration 0.59 ± 0.06 years), and at the second follow-up (mean duration 5.15 ± 1.25 years). Fasting serum samples collected from CSs and patients started in June 2009 and the last participant was recruited in November 2014. The 5-year follow-up serum sample gathering began in May 2013 and ended in November 2017, and only comprised the patients’ group. Serum samples were collected using the standard antecubital venipuncture technique between 09:00 and 11:00 a.m. Blood (5 mL) was sampled in anticoagulant-free tubes and kept for 1 h at 4 °C (for platelet activation). The blood was subsequently centrifuged at 2000× *g* for 15 min at 4 °C, and the serum was aspirated, divided into aliquots, and immediately frozen and stored at −20 °C for up to 2 weeks or at −80 °C for longer periods.

We used the Brief Psychiatric Rating Scale (BPRS) [33] to assess the presence of psychopathological symptoms in patients. The BPRS consists of 18 symptoms and each item is measured on a seven-point Likert scale from “not present” to “extremely severe”. A total score was used as the outcome. Fasting blood samples and BMI data from CSs were collected cross-sectionally. All participants records were handled according to the confidentiality practices. Samples were anonymized and sequentially numbered. A lab coding system and database were developed for recording anonymized participant and sample information. Study data were collected and managed using REDCap electronic data capture tools hosted at the University of Tartu, Estonia [34,35].

### 2.3. Measurement of Metabolites

#### 2.3.1. eCBs and eCB-like Compounds Quantification

Fifty µL of serum was mixed with 10 µL of internal standard (amino acid standard set A, Cambridge Isotope Laboratories, USA; [^2^H_8_]arachidonic acid, Cayman Europe, Estonia) and extracted by addition of 750 µL of ice-cold methanol. After 10 min incubation at −20 °C, the samples were centrifuged for 10 min 21,500× *g*. The supernatants were transferred into new vials and dried under a stream of nitrogen. The samples were resolved in 80 µL of methanol with 0.2% formic acid. Twenty µL was injected into high-performance liquid chromatography (Agilent 1200 series, Waldbronn, Germany)–mass spectrometry (Sciex Q-Trap 4500, Framingham, MA, USA) analysis on C18 column (Kinetex 2.6 µm EVO C18 100 × 4.6 mm, Phenomenex, Torrance, CA, USA). The samples were analyzed with a flow rate of 400 µL/min, 2 min 2% acetonitrile in water, 12 min 100% acetonitrile, and isocratic acetonitrile until 25 min. All solvent contained 0.2% formic acid. The retention time and optimal ionization/fragmentation condition for each compound were determined with commercial standards. The selected reaction monitoring transitions were 2-AG 379/287, AEA 348/62, LEA 324/62, and OEA 326/62, PEA 300/62. Electrospray ionization was performed at 500 °C. For quantification, standard curves from known concentrations of commercial compounds and constant amounts of internal standards were created. For each analysis patch of 18 samples, a quality control (mixture of 20 randomly chosen samples from each study group) and a blank were included.

Due to the use of serum, it was, however, not possible to differentiate between the levels of 1-AG and 2-AG because of their rapid isomerization at room temperature. Based on a previous study [36], we assumed that 1-AG was predominantly derived from 2-AG. Therefore, identified by a chromatographic method, we treated the total AG concentration as 2-AG.

#### 2.3.2. PCs Quantification

To assay serum levels of PCs, we applied the AbsoluteIDQ™ p180 kit (BIOCRATES Life Sciences AG, Innsbruck, Austria) using the flow injection analysis tandem mass spectrometry (MS) (QTRAP 4500, Sciex, Framingham, MA, USA), as well as liquid chromatography (Agilent 1260, Waldbronn, Germany) technique, according to the manufacturer’s protocol. The AbsoluteIDQTM p180 kit allows for simultaneous quantification of PCs (PC aa = diacyl) amongst other metabolites. Lipid side chain composition is described as Cx:y, where x denotes the number of carbons in the side chain and y denotes the number of double bonds, although the measurement did not allow for differentiation of PCs on the bond type level.

### 2.4. Statistical Analyses

All data were checked for normality of distribution using the Shapiro–Wilk test. Normally distributed data (age, weight, BMI, and waist circumference) were analyzed using the Student’s *t*-test or repeated measure ANOVA, and mean differences were tested with Scheffé post hoc test. Dichotomous data (gender and smoking status) were analyzed using the chi-square test. To examine the alterations of putative biomarker levels between CSs and FEP patients over time, linear mixed-effects (LME) models were used. Repeated measurements were handled by including a random intercept for participants in the model (since patients have different baseline values) and by allowing for time-dependent correlations between different measurements of each patient. The model intercept was set to represent putative biomarker levels of the control group and variables’ coefficients were compared to this intercept. Each set of analysis was adjusted for potential confounders: gender, age at the first visit, smoking status, and the time difference between the visits (time difference between expected time and given time). However, these covariables were not of primary interest in this study. Time dependence between measurements of each patient was modeled by continuous autoregressive correlation structures of order 1 and models were fitted by maximum likelihood method. The candidate biomarker data were log-transformed before analysis to reduce the heterogeneity of variance commonly seen with metabolic data. Moreover, several metabolite ratios were calculated as indicators of metabolic processes.

First, to identify dependent variables, which behave differently in the case of patients and the control group, we fitted two nested models to the data (i.e., reduced model with dependent variables with no patients’ specific independent variables compared to a more complex model with added terms allowing for the possibility for the value of the dependent variable to depend on patients’ type of visit and time between visits). We compared models using the likelihood ratio test, where the false discovery rate (FDR) procedure was implemented for multiple testing corrections [37], setting a cut-off of <0.005 [38]. Thereafter, the estimates from the LME analyses (fixed effects) were used to establish patients’ biomolecule profile alterations at three different time points. As we ran several LME models in parallel, and the selected metabolites belonged to classes of lipids that may share partially similar biosynthetic pathways and the correlation within the same lipid class is high, we considered the adjustment of the significance level to *p* ≤ 10^−4^ while selecting the metabolites with the most pronounced change, and the *p*-values between <0.005 and 10^−4^ are referred to as a trend change.

The R [39] statistical language version 3.5.2 package *nlme* [40] and ANOVA-type diagnostic test were used to perform analysis of the relationship between candidate biomarker levels differences among CSs and patients at three time points, and Statistica software for Windows [41] was used for other analyses. The visualization of error variances obtained from regression results was computed using R software package *ggplot2* [42]. Residuals or errors terms were assumed to be independent among individuals but dependent within each participant.

## 3. Results

### 3.1. General Description of the Study Samples

The demographic and clinical characteristics of the study participants are shown in Table 1. There were no statistically significant differences between AP-naïve FEP patients and CSs in terms of their age (t_(110)_ = 1.87, *p* = 0.07), gender (χ^2^_(1)_ = 3.58, *p* = 0.06), or mean values of BMI (t_(110)_ = 0.34, *p* = 0.74). In the patient group, the AP treatment reduced the psychopathology (BPRS) score significantly (*p* < 10^−6^) but caused a significant increase in BMI (*p* < 10^−6^). The patients’ average length of education in years was significantly (*p* < 10^−5^) lower compared to that of the CSs.

### 3.2. Biomolecule’s Alterations and BMI Change in Patients

**Figure 1:**
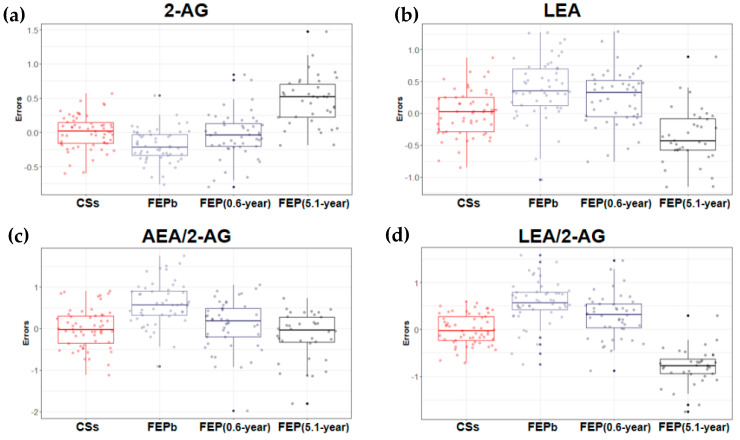

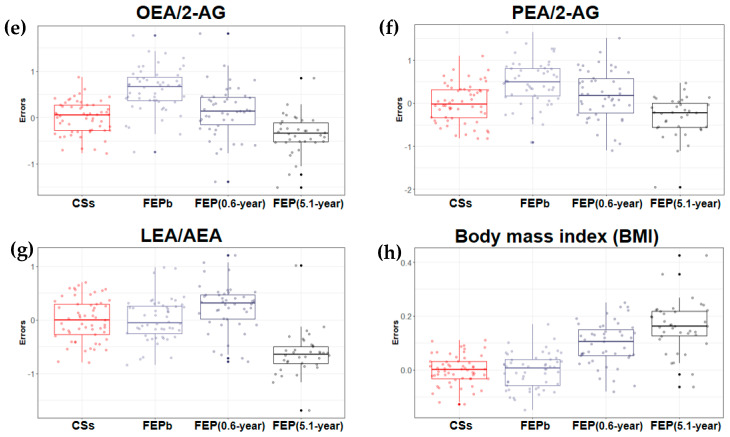
Boxplots of the variation of prediction errors of log-transformed levels of eCB, eCB-like compound, their ratios (**a**–**g**), and BMI (**h**) (derived by regressing out covariate effects) for control subjects (CSs) and first-episode (FEP) patients at baseline (FEP_b_, before treatment with antipsychotics (AP)), after 0.6-year (FEP_(0.6-year)_), and after 5.1-year (FEP_(5.1-year)_) treatment with AP. The solid horizontal line in each box represents the median. The area above and below the line represents the 50th to the 75th and the 25th to the 50th percentiles, respectively. The whiskers extend to the highest and lowest values contained within 1.5 times the interquartile range of the data. Each calculated error is represented as a dot.

Having collected a set of multivariate data, the first step was to test the effects of the AP treatment and disease status by LME models based on 2-AG, AEA, LEA, OEA, PEA, and their metabolically dominating specific lipids: 38 PCs (PC aa C24:0—PC aa C42:6), and BMI. The patients’ data were compared to CSs after adjusting for covariates. For the primary analysis, a set of two LME models was tested; both models used all the available data, but patient-specific determinants were only considered in the unrestricted models. Details of the models considered are given in Supplementary Materials, Table S1. These results justified conducting further LME regression models involving selected covariates to test the objectives of the study. Concerning the serum levels of eCBs (2-AG, AEA) and eCB-like NAEs (LEA, OEA, and PEA), the concentrations of AEA (t_(72)_ = 3.03, *p* = 0.003), LEA (t_(72)_ = 3.17, *p* = 0.002), OEA (t_(72)_ = 3.84, *p* = 3 × 10^−4^ and PEA (t_(72)_ = 3.36, *p* = 0.001) tended to be elevated in the AP-naïve patients’ group compared to the CSs, with an opposite trend for a concentration of 2-AG (t_(76)_ = −2.97, *p* = 0.004). More prominent alterations in the function of the ECS emerged when we compared the ratios of AEA/2-AG, eCB-like NAEs, and 2-AG between the groups. The ratio levels of AEA/2-AG, LEA/2-AG, OEA/2-AG, and PEA/2-AG were significantly elevated (t_(72)_ = 4.67, t_(72)_ = 4.38, t_(72)_ = 5.24, t_(72)_ = 4.95, respectively, *p* < 10^−4^ ) among the AP-naïve patients. Meanwhile, the ratio levels of LEA/AEA, OEA/AEA, and PEA/AEA and the BMI values did not differentiate the patients and CSs at this time point (the results are summarized in Table 2, and significant differences are depicted in Figure 1).

Data for eCBs and eCB-like NAEs after the 0.6-year AP treatment showed that all of the altered metabolites and their ratio levels returned to levels comparable with those of the CSs. At the same time, the treatment had increased patients’ BMI (t_(77)_ = 3.59, *p* = 6 × 10^−4^ ).

In the fifth year of follow-up, previously downregulated 2-AG was considerably elevated (t_(76)_ = 5.34, *p* < 10^−4^ ), and previously elevated ratios of LEA/2-AG, as well as LEA/AEA, were significantly lowered (t_(72)_ = −4.87, *p* < 10^−4^ , (t_(72)_ = −4.65, and *p* ≤ 10^−4^ respectively). Moreover, patients gained further weight during the continuous follow-up period, with a statistically significant increase in BMI (t_(77)_ = 4.63, *p* < 10^−4^ ).

Thereafter, for a better overview of the disease-related eECS functioning alterations, we also analyzed PCs serum concentration differences between the groups and within the patients’ group. A total of 38 lipids were analyzed, among which, 15 were significantly different between AP-naïve patients and CSs. Six lipids were significantly ((*p* ≤ 10^−4^ ) increased in the patients’ group, namely, PC aa C40:2 (t_(74)_ = 5.29), PC aa C40:3 (t_(74)_ = 4.94), PC aa C42:0 (t_(74)_ = 4.02), PC aa C42:1 (t_(74)_ = 5.10), PC aa C42:2 (t_(74)_ = 5.26)), and PC aa C42:4 (t_(74)_ = 4.57). A similar tendency was found for PC aa C36:0 (t_(74)_ = 3.52, *p* = 7 × 10^−4^ ) and PC aa C40:1 (t_(74)_ = 3.86, *p* = 2 × 10^−4^ ). At the same time, levels of PC aa C32:2 (t_(74)_ = −5.92), PC aa C34:2 (t_(74)_ = −4.27), PC aa C34:3 (t_(74)_ = −5.54), PC aa C34:4 (t_(74)_ = −7.05), PC aa C36:1 (t_(74)_ = −4.12), PC aa C36:2 (t_(74)_ = −6.83), PC aa C36:3 (t_(74)_ = −7.64), PC aa C38:3 (t_(74)_ = −4.67), PC aa C38:5 (t_(74)_ = −4.05), and PC aa C38:5 (t_(74)_ = −4.05) were significantly (*p* ≤ 10^−4^ ) more reduced in the AP-naïve patients’ group than those in the CSs. The same directional trends emerged for PC aa C30:0 (t_(74)_ = −3.44, *p* = 0.001), PC aa C36:4 (t_(74)_ = −3.78, *p* = 3 × 10^−4^ ), PC aa C36:5 (t_(74)_ = −3.56, *p* = 7 × 10^−4^ ), PC aa C36:6 (t_(74)_ = −3.60, *p* = 6 × 10^−4^ ), PC aa C38:4 (t_(74)_ = −3.78, *p* = 3 × 10^−4^ ), PC aa C40:5 (t_(74)_ = −3.70, *p* = 4 × 10^−4^ ), and PC aa C40:6 (t_(74)_ = −3.67, *p* = 5 × 10^−4^ ) (full details of the data generation and fitting procedures are given in Supplemental Table S2).

Seven months of AP treatment significantly improved pre-existing shifts in serum PCs levels. However, the level of PC aa C42:2 (t_(74)_ = 4.04, *p* = 10^−4^ ) was still significantly elevated and PC aa C36:0, PC aa C40:1, PC aa C40:2, PC aa C40:3, PC aa C42:1, and PC aa C40:4 maintained the same directional change as previously described (see Supplementary Table S2 for further details).

Five years of SSD and AP treatment revealed significant changes in the measured lipid precursors in 16 out of 38 cases and, additionally, trends in alterations emerged for four PCs. The concentrations of PC aa C30:2 (t_(74)_ = −5.11), PC aa C36:0 (t_(74)_ = −4.40), PC aa C40:1 (t_(74)_ = −4.76), PC aa C40:2 (t_(74)_ = −7.51), PC aa C40:3 (t_(74)_ = −7.10), PC aa C40:4 (t_(74)_ = −4.59), PC aa C42:0 (t_(74)_ = −4.33), PC aa C42:1 (t_(74)_ = −6.31), PC aa C42:2 (t_(74)_ = −7.40), PC aa C42:4 (t_(74)_ = −7.85), PC aa C42:5 (t_(74)_ = −4.76), and PC aa C42:5 (t_(74)_ = −4.69) were significantly (*p* ≤ 10^−4^ ) lower than those in the CSs. In addition, the downward trend emerged for PC aa C32:3 (t_(74)_ = −3.71, *p* = 4 × 10^−4^ ). In contrast, PC aa C38:1 (t_(74)_ = 4.47), PC aa C38:5 (t_(74)_ = 4.67), PC aa C38:6 (t_(74)_ = 4.62), and PC aa C40:6 (t_(74)_ = 5.21) were significantly (*p* ≤ 10^−4^ upregulated and the same directional trends emerged for PC aa C36:5 (t_(74)_ = 3.33, *p* = 0.001), PC aa C38:4 (t_(74)_ = 3.69, *p* = 4 × 10^−4^ ), and PC aa C40:5 (t_(74)_ = 3.85, *p* = 3 × 10^−4^ (see Supplementary Table S2). In addition, significant serum concentration differences (expressed in terms of residuals) in each measured PC between the CSs and patients over three different time points are represented by box plots, as shown in Supplementary Figure S1.

The results indicate distinctions in the lipid metabolic network and functionality in patients with SSD that occurs early in the development of the disease and during the continuation of the disease and its treatment with APs.

The figures comprise only those analyzed characteristics for which the effect of the treatment and the effect of the disease duration *F*-value was > |8|, according to the linear mixed-effects models.

## 4. Discussion

A plethora of scientific literature demonstrates that changes in lipid composition might be associated with the onset and progression of various psychiatric diseases, including SSD. Our metabolic study describes, for the first time to our knowledge, a dysregulation of the eECS components in patients who have suffered the FEP, during the 5-year follow-up. While interpreting our results, we focused more on the metabolic shifts associated with the disease onset and the 5-year AP treatment because the 0.6-year treatment eliminated or significantly reduced the shifts that had occurred at the disease onset.

According to our results, the level of 2-AG tended to be decreased in AP-naïve FEP patients, and 5 years of AP treatment resulted in a significant increase in 2-AG. There is, however, little information on the status of 2-AG in psychotic disorders [43]. In contrast, the levels of AEA, LEA, OEA, and PEA tended to be elevated in AP-naïve FEP patients, resulting in a significant increase in ratios of AEA/2-AG, LEA/2-AG, OEA/2-AG, and PEA/2-AG. After five years, the levels of NAEs were comparable to the levels of NAEs in the controls, and the ratios of LEA/2-AG and LEA/AEA had decreased. Recently, Potvin et al. [44] also demonstrated that the blood levels of AEA and OEA were increased in acutely decompensated schizophrenia patients and that AP treatment normalized these alterations. However, some studies reported no change in the serum AEA level in drug-naïve FEP patients [45,46] and lower levels of serum PEA in AP-naïve FEP patients compared to CSs [47]. Discrepancies between our study and some earlier studies may be due to methodological differences or due to differences between the study population, such as the diagnosis or use of psychotropic drugs. To the best of our knowledge, no previous studies on serum LEA levels have been published in AP-naïve FEP or treated SSD patients, but our results emphasize that LEA is important alongside AEA, PEA, and OEA when examining SSD-related alterations in the eECS.

The cannabinoid receptors, eCBs, and their synthesizing and metabolizing enzymes are presented in the central and peripheral nervous systems and many other peripheral tissues [48,49]. AEA and 2-AG bind to both CB_1_ and CB_2_ receptors, but with different responses, efficacy, and specificity [49]. As an abundantly formed intermediate in the lipid metabolism pathways, 2-AG is a complete agonist at both the CB_1_ and CB_2_ receptors [50,51]. Additionally, AEA and 2-AG can act on PPARs through either direct or indirect pathways: AEA activates both PPAR-α and PPAR-γ, and 2-AG prefers PPAR-γ [15,52]. When activated by its ligands, PPARs join to specific DNA response units to regulate gene transcription and expression, and are therefore involved in the modulation of lipid and glucose metabolism, promotion of cell differentiation, and inflammation [15,52]. Importantly, the 2-AG level is up to 170 times higher than the AEA in the brain [50], suggesting that 2-AG may be physiologically more important than AEA [53]. AEA behaves as a partial agonist at both the CB_1_ and CB_2_ receptors [51,54]. Following the CB_1_ and CB_2_ receptor activation by their agonists, multiple intracellular signal transduction pathways are triggered. CB_1_ receptors are predominantly found in the gamma-aminobutyric acid (GABA) and glutamatergic neurons, possessing an excitatory or inhibitory activity. In addition, cannabimimetic actions over CB_1_ receptor activation are associated with analgesia, neuroprotection, hypotension, and appetite stimulation [55]. CB_2_ receptors are very abundant in immune tissues and cells, and they are primarily expressed only in active inflammation [48,56]. In addition, eCBs effects are not restricted to the CB receptors [48]. For instance, AEA binds to the TRPV1 receptors, upon which, it acts as a full agonist [57]. The ion channel TRPV1 is involved in nociception, thermosensation, energy homeostasis, etc. In the interaction with the ECS, it affects energy homeostasis, either in regulating food consumption through controlling appetite or energy expenditure through producing heat [58]. It is worth emphasizing that most eCB-like NAEs levels are up to 10 times higher than AEA levels [12] and exert a variety of bioactions. OEA is a high-affinity agonist of the PPAR-α, which triggers the FAs uptake and upregulation of genes associated with FAs transport and peroxisomal or mitochondrial FAs beta-oxidation, particularly under conditions of a lipid-rich diet and fasting-induced lipolysis [59]. Clinically, PPAR-*α* agonists, which include fibrates, are used in the treatment of cholesterol disorders and for their effects on several cardiovascular risk markers associated with the metabolic syndrome and diabetes [60]. PEA shows anti-inflammatory, analgesic, and neuroprotective actions through directly activating multiple different receptors: PPAR-α and GPR55 [61,62,63]. Moreover, PEA indirectly activates CB1, CB2, and TRPV1 receptors by inhibiting the degradation of AEA [60,64]. Thus, OEA and PEA may also play a significant role in the regulation of the cardiometabolic risk associated with type 2 diabetes and obesity [65]. PPAR-α seems to display some preference for NAEs, such as PEA, OEA, and LEA [66]. It has been suggested that LEA found within the intestine exhibits anorectic effects via the activation of PPAR-α in the enterocytes followed by the activation of afferent vagal fibers leading to the brain [66]. Additionally, OEA also exerts satiety-inducing effects by activating the hedonic dopamine pathways [59]. Furthermore, OEA, as well as LEA, are endogenous ligands for GPR119 whose activation leads to a reduction in both food intake and body weight gain, as GPR119 agonists secure glucose control by directly enhancing insulin secretion in pancreatic β-cells and by stimulating the secretion of gut hormones, including glucagon-like peptide-1 (GLP-1), which, in turn, induce additional increases in insulin secretion and improve hepatic glucose metabolism [67].

Humans utilize mainly dietary-derived carbohydrates, lipids, and proteins as sources of energy. Excessive dietary intake leads to the accumulation of body fat in the form of triglycerides produced by the overconsumption of fats and carbohydrates. Recently, Smith et al. [68] demonstrated that AP treatment increases the subcutaneous and visceral adipose tissue size among patients with SSD, and young AP-naive patients may be particularly susceptible to this effect. In addition, Pillinger et al. [69] confirmed that marked differences exist between APs in terms of their risk of causing metabolic side-effects. Although AP treatment could contribute to this, several studies have found evidence for glucose and lipid alterations at the illness onset. It has been suggested that patients with SSD might be predisposed to metabolic dysregulation in terms of an increased incidence of insulin resistance, fasting glucose levels, impaired glucose tolerance, and dyslipidaemia, even without exposure to AP drugs, due to intrinsic disease links [70,71]. Our results showed that the onset of the FEP is associated with a metabolic shift toward energy expenditure (increased serum levels of NAEs, decreased 2-AG levels, and increased ratios’ values between these biomarkers). We theorize that, at this stage of the disease, the metabolic response to stress manifests through the increased signalling through the PPAR-α receptor and decreased signalling through the CB_1_ receptors. Further, 0.6-years of AP treatment eliminated these changes. Five years of the disease with a concomitant AP treatment revealed shifts in the metabolism toward energy storage through the activation of CB_1_ receptors because of significantly elevated levels of 2-AG. The orexigenic effects associated with the peripherally increased level of 2-AG are manifested by the activation of hepatic CB1 receptors, which induces the expression of the lipogenic transcription factor SREBP1c and its associated lipogenic enzymes, acetyl CoA carboxylase, and FA synthase, which are major enzymes for FA synthesis [72]. The result is a positive energy balance, and thus the development of obesity, as evidenced by a significant increase in BMI in the patients’ group over the 5-year follow-up. Extensive research focused on the central and peripheral role of the eECS in the energy balance [73], and it is convincingly shown that the controlled downregulation of the CB_1_ receptors and upregulation of PPAR-α and PPAR-γ are involved in the therapeutic effects exhibited by eCB-s and eCB-like NAEs in the role of the regulation of lipid metabolism and obesity [74,75]. According to interdisciplinary studies, PPAR-γ agonists may reverse the brain energetic signature in SSD and help to restore the bioenergetic abnormalities among a subgroup of patients with cognitive dysfunction [76], and Wada et al. [77] reported evidence of the causal relationship between the dysfunction of PPAR-α and SSD. They demonstrated that the mechanisms underlying SSD pathogenesis involve PPAR-α regulated transcriptional machinery and the modulation of synapse physiology. In addition, influencing these receptor targets has been shown to have a neuroprotective effect [78,79]. Collectively, PPAR agonists would also have a high potential in the management of SSD treatment. Thus, our results, in line with other studies, support the concept that the eECS functioning is in an altered state in SSD patients, and that a specific pattern of disturbing activity during the different stages of the disease is associated with a dysregulation in systemic energy metabolism, presumably through CB receptors and PPARs.

Moreover, to validate our longitudinal, complex, and dynamically time-altered results at the eCBs and eCB-like NAE levels, we also examined their possible precursors. Analyses of PCs supported our previously described results. Regarding the elevated serum levels of NAEs in the AP-naïve group, we detected that the levels of their respective precursor FAs (i.e., PC aa C36:0, PC aa C38:1, PC aa C40:1-C40:4, and PC aa C42:0-C42:5) were significantly increased. In other words, the serum levels of these PCs followed the levels of NAEs. Thus, we may assume that the precursors were used intensively to produce (among other biomolecules) NAEs from them. In contrast, the decrease in 2-AG was accompanied by significantly reduced levels of the respective precursors to 2-AG (PC aa C34:4, PC aa C36:4-C36:5, PC aa C38:4-C38:6, and PC aa C40:5-C40:6) among AP-naive patients. The mentioned PCs dominated in the cell membranes typically with arachidonic acid (C20:4) at the sn-2 position and the saturated or unsaturated FAs (i.e., C16:0, C18:0, C18:1 or C18:2) at the sn-1 position. Membrane PLs were subjected to turnover by phospholipases and transacylases as part of lipid acyl chain remodeling or degradation. It can also be said that these PCs serum levels were similar to the levels of NAEs, on the one hand, and the levels of 2-AG on the other. Our results emphasize the activation of compensatory mechanisms involving whole-body homeostasis upon FEP onset. Furthermore, we showed that changes were partially or completely resolved after 0.6 years of AP treatment. However, by the fifth year, the situation was the opposite of FEP, based on the duration of the disease and continuous AP treatment. The levels of PC aa C36:0, PC aa C38:1, PC aa C40:1-C40:4, and PC aa C42:0-C42:5 were significantly reduced and, as a consequence, fewer NAEs were synthesized. Therefore, we may speculate that the activation of the PPAR-α receptors was scarce and led to increased FA beta-oxidation, increased tissue deposition of FAs, and intensified inflammatory processes in the fifth year of the disease and AP treatment. Furthermore, concerning the elevated level of 2-AG, our results also showed that the levels of possible precursors PC (e.g., C34:4, PC aa C36:4-C36:5, PC aa C38:4-C38:6, and PC aa C40:5-C40:6) were significantly increased. These results support the hypothesis that SSD patients have a disturbed FA metabolism characterized by shifts in lipid precursors for eCBs and eCB-like compounds, which may reflect compositional and functional alterations of cell membranes.

In the light of previous research, the study of the eECS in different stages of SSD is of interest for at least two main reasons. First, previous studies have confirmed close interactions between the peripheral and central ECS, suggesting that peripheral serum levels may reflect concentrations of eCBs and eCB-like NAEs found in the brain. In addition, it is shown that serum eCB levels relate to the severity of SSD symptoms, albeit the results are contradictory [26,44]. We admit that our results allow us to conclude primarily about the functionality of the peripheral eECS. Secondly, SSD is a chronic but highly heterogeneous disease, and diversity in the disease progression or response to APs is not limited to the characteristics of time-varying brain function, or is associated only with the classical monoamine neurotransmitter systems. We must keep in mind that, in addition to SSD-specific symptoms, the eECS modulates a very complex network of organ-, tissue-, and cell-specific metabolic and inflammatory signalling pathways, which affect the brain and the whole body. Our study suggests the existence of alterations in the peripheral serum levels of eCBs and eCB-like NAEs in AP-naïve FEP patients. Alterations of these endogenous mediators may suggest a protective role of increased ECS signalling in maintaining homoeostasis under an acute psychotic state [46,47]. In other words, under acutely stressful conditions, as in most cases, SSD is triggered by major stress or a traumatic event. Previously, Dlugos et al. [80] showed that AEA, OEA, and PEA blood concentrations increase immediately after the acute stress period, and Hill et al. [81] demonstrated that FA amides are higher in individuals who show stress tolerance. In contrast, studies focusing on the cumulative burden of chronic stress and negative life events have shown that the long-term overstimulation of the hypothalamic–pituitary–adrenal (HPA) axis leads to an increase in 2-AG [82], which is associated with harmful consequences for mental health [81]. According to the results of our study, the progression of the disease and concomitant 5-year AP treatment reflect the development of chronic stress in the body, which was indirectly confirmed by a significant increase in 2-AG and a decrease in NAEs levels. However, despite the existing evidence suggesting that the HPA axis and the ECS may have a role in psychosis, the precise nature of these relationships is unclear [83].

In addition, it is widely documented that eCBs and eCB-like NAEs have a major role in the suppression of low-grade chronic inflammation through multiple pathways [84]. Similar to a previous report [78], we speculate that increased levels of PEA and AEA and a decreased level of 2-AG found in the AP-naïve group, as well as the considerably elevated level of 2-AG during the 5-year follow-up in our study, might be a consequence or the causative factors of the immunomodulatory effect of the eECS. In addition, various studies have shown that, depending on the context, these bioactive molecules can perform anti- and pro-inflammatory actions. In particular, PEA and AEA demonstrate mainly anti-inflammatory effects, whereas 2-AG exhibits pro-inflammatory and anti-inflammatory functions [85,86]. Furthermore, this hypothetical mechanism would also explain why the 7-month AP treatment lowered eCBs levels back to those observed in CSs, since APs could have anti-inflammatory effects in FEP, confirmed by a recently published meta-analysis [81], and also earlier by us [30,87,88]. However, the positive immunomodulatory effect of APs does not persist over time, as patients often develop a metabolic disorder with a significant increase in BMI. We observed the same trend in our study. Further studies are needed to identify whether the over-activation of the eECS at the fifth year of SSD and continuous AP treatment is an ineffective adaptive response to restore homeostasis or a maladaptive mechanism that contributes to disease progression.

Several potential limitations of our study require consideration. First, the samples sizes are relatively small, although large enough to enable the detection of changes in the levels of biomolecules [89]. Second, we collected data from CSs at one point in time. Third, based on the naturalistic study design, the choice or the dosage regimen of AP after enrolment and during the 5-year follow-up period did not have any descriptions. Therefore, we were not able to determine the effects of specific active ingredients on biomolecule levels. Third, due to the naturalistic study design, psychiatrists had the flexibility to choose which AP drug to use to treat the patients and at what dose, and to change treatment regimens if necessary. Before the follow-up study visits, patients were not required to take certain medications for a fixed period, according to a standard schedule. We provided information in terms of the CPZE doses of the APs, to describe the need for therapeutic doses that mainly alleviate the positive symptoms of the SSD. However, these calculated doses are based on consensus estimates that describe how the drug binds to D2 receptors. However, the metabolic side effects associated with AP treatment are caused by drug molecules (mostly) binding to alternative receptors. Therefore, we could not demonstrate a direct therapeutic dose impact of the APs on the levels of biomolecules.

## 5. Conclusions

In this study, we provide valuable insight into the altered function of the eECS at the time of FEP onset and during the disease progression in the context of AP treatment. Our results suggest that FEP might be characterized, among other factors, by an elevation in the peripheral concentration of AEA, LEA, OEA, and PEA and by decreased 2-AG levels, while AP-naïve patients are compared to CSs. Our findings indicated that the NAEs-mediated upregulation of PPAR-α receptors manifested itself in energy expenditure. These changes are at least partially reversed by the 0.6-year AP treatment. Further, the 5-year monitoring of the patients’ disease progression and the therapeutic effect led to an increase in 2-AG levels, which, at the same time, contributed to enhanced CB_1_ receptor-mediated effects, which manifested in obesity. Our results confirmed that dynamic 2-AG, NAEs, and their possible lipid precursors in terms of PCs, are relevant to the description of the metabolic shifts resulting from the altered eECS function during and after FEP.

## Figures and Tables

**Table 1:** Characteristics of control subjects (CSs) and first-episode psychosis (FEP) patients at baseline (before treatment with antipsychotics (FEP_b_), after 0.6-year treatment (FEP_0.6-year_), and after 5.1-year treatment (FEP_5.1-year_) with antipsychotics.

*Characteristics*	Participants				Comparison between Groups	
	CSs	FEP_b_	FEP_(0.6-year)_	FEP_(5.1-year)_	FEP_b_ and CSs	FEP_b,_ FEP_(0.6-year)_ and FEP_(5.1-year)_
Participants	58	54	47	38		
Age (years), mean ± SD (range)	24.7 ± 4.5 (19.1–39.3)	26.6 ± 6.1 (18.7–41.1)	27.3 ± 6.4 (19.3–41.7)	31.8 ± 5.9 (23.7–46.2)	t_(110)_ = 1.87 ns	-
Men (%)	24 (44%)	31 (57%)	27 (51%)	23 (43%)	χ^2^_(1)_ = 3.58 ns	-
Current cigarette smoker (n,%)	15 (26%)	18 (33%)	16 (30%)	20 (37%)	χ^2^_(1)_ = 0.33 ns	-
BMI (kg/m^2^), mean ± SD (range)	22.6 ± 2.8 (16.8–28.9)	22.8 ± 3.0 (18.4–30.2)	25.3 ± 3.9 ^a^ (18.8–34.7)	27.8 ± 4.7 ^b,c^ (18.8–43.0)	t_(110)_ = 0.34 ns	*F*_(2)_ = 19.26 *p* < 10^−6^
BPRS score mean ± SD (range)	-	49.9 ± 15.4 (13–85)	22.9 ± 12.7 ^a^ (2–48)	14.2 ± 10.5 ^b,c^ (0–49)	-	*F*_(2)_ = 93.6 *p* < 10^−6^
AP dose mean ± SD (range)	-	-	365 ± 163 (60–680)	442 ± 297 (75–1566)	-	t_(52)_ = −0.36 ns
Length of education mean ± SD (range)	14.6 ± 1.8 (10.0–19.0)	12.6 ± 2.5 (8.0–18.0)	-	-	t_(109)_ = −4.86 *p* < 10^−5^	-

BMI—body mass index; BPRS—Brief Psychiatric Rating Scale; AP dose—chlorpromazine equivalent dose of antipsychotics; ^a^—statistically significant difference (*p* < 0.05) between patients before (FEP_b_) and after 0.6-year treatment (FEP_(0.6-year)_); ^b^—statistically significant difference (*p* < 0.05) between 0.6-year (FEP_(0.6-year)_) and 5.1-year treatment (FEP_(5.1-year)_); ^c^—statistically significant difference (*p* < 0.05) between patients before (FEP_b_) and after 5.1-year treatment (FEP_(5.1-year)_); ns—not significant (*p* ≥ 0.05).

**Table 2:** Estimated effects of body mass index, serum concentrations of endocannabinoids, endocannabinoids-like compounds and their ratios between control subjects (CSs, *n* = 58), first-episode psychosis patients at baseline (before treatment with antipsychotics, FEP_(b)_, *n* = 54), after 0.6-year treatment (FEP_(0.6-year)_, *n* = 47), and after 5.1-year treatment (FEP_(5.1-year)_, *n* = 38) with antipsychotics (results from the linear mixed-effects model).

	Intercept	Age	Gender	Smoking	Disease and Treatment Effect			TimeDiff1	TimeDiff2
					FEP Patients before Treatment	FEP Patients after 0.6-Year Treatment	FEP Patients after 5.1-Year Treatment		
	Effects of Independent Variables on the Dependent Variable (*F*-Value, *p*-Value)								
					t-Value, *p*-Value				
**2-Arachidonoylglycerol** **(2-AG)**	***F*_(1,110)_ = 81.02,** ***p* < 10^−4^**	***F*_(1,76)_ = 1.41,** ***p* = 0.24**	***F*_(1,110)_ = 0.46,** ***p* = 0.50**	***F*_(2,76)_ = 1.89,** ***p* = 0.16**	***F*_(3,76)_ = 25.19, *p* < 1 × 10^−4^**			*F*_(1,76)_ = 0.72, *p* = 0.40	*F*_(1,76)_ = 5.97, *p* = 0.02
					t_(76)_ = −2.97, *p* = 0.004	t_(76)_ = −0.34, *p* = 0.73	**t_(76)_ = 5.34,** ***p* < 10^−4^**		
Anandamide (AEA) (*N*-arachidonoylethanolamine)	*F*_(1,110)_ = 12.59, *p* = 6 × 10^−4^	*F*_(1,72)_ = 2.59, *p* = 0.11	*F*_(1,110)_ = 0.07, *p* = 0.79	*F*_(2,72)_ = 0.80, *p* = 0.45	*F*_(3,72)_ = 4.62, *p* = 0.005			*F*_(1,72)_ = 5.08, *p* = 0.03	*F*_(1,72)_ = 2.11, *p* = 0.15
					t_(72)_ = 3.03, *p* = 0.003	t_(72)_ = 0.48, *p* = 0.64	t_(72)_ = 1.59, *p* = 0.12		
**Linoleoylethanolamide (LEA)** (C18:2, *N*-cylethanolamine)	*F*_(1,109)_ = 32.48, *p* < 10^−4^	*F*_(1,72)_ = 0.39, *p* = 0.54	*F*_(1,109)_ = 2.39, *p* = 0.13	*F*_(2,72)_ = 0.76, *p* = 0.47	***F*_(3,72)_ = 15.11, *p* < 10^−4^**			*F*_(1,72)_ = 0.38, *p* = 0.54	*F*_(1,72)_ = 2.59, *p* = 0.11
					t_(72)_ = 3.17, *p* = 0.002	t_(72)_ = 1.97, *p* = 0.05	t_(72)_ = −2.62, *p* = 0.01		
Oleoylethanolamide (OEA) (C18:1, *N*-acylethanolamine)	*F*_(1,109)_ = 136, *p* < 10^−4^	*F*_(1,72)_ = 2.94, *p* = 0.09	*F*_(1,109)_ = 0.005, *p* = 0.95	*F*_(2,72)_ = 0.54, *p* = 0.58	*F*_(3,72)_ = 6.63, *p* = 5 × 10^−4^			*F*_(1,72)_ = 2.46, *p* = 0.12	*F*_(1,72)_ = 1.47, *p* = 0.23
					t_(72)_ = 3.84, *p* = 3 × 10^−4^	t_(72)_ = 0.98, *p* = 0.33	t_(72)_ = 0.52, *p* = 0.60		
Palmitoylethanolamide (PEA) (C16:0, *N*-acylethanolamine)	*F*_(1,110)_ = 251, *p* < 10^−4^	*F*_(1,71)_ = 1.91, *p* = 0.17	*F*_(1,110)_ = 0.01, *p* = 0.90	*F*_(2,71)_ = 0.95, *p* = 0.39	*F*_(3,71)_ = 4.36, *p* = 0.007			*F*_(1,71)_ = 2.64, *p* = 0.11	*F*_(1,71)_ = 3.84, *p* = 0.05
					t_(71)_ = 3.36, *p* = 0.001	t_(71)_ = 1.51, *p* = 0.14	t_(71)_ = 0.97, *p* = 0.33		
**AEA/2-AG**	*F*_(1,110)_ = 73.88, *p* < 10^−4^	*F*_(1,72)_ = 1.18, *p* = 0.28	*F*_(1,110)_ = 0.02, *p* = 0.88	*F*_(2,72)_ = 2.52, *p* = 0.09	***F*_(3,72)_ = 13.95, *p* < 10^−4^**			*F*_(1,72)_ = 4.11, *p* = 0.05	*F*_(1,72)_ = 0.09, *p* = 0.76
					**t_(72)_** **= 4.67,** ***p* < 10^−4^**	t_(72)_ = 0.79, *p* = 0.43	t_(72)_ = −1.10, *p* = 0.28		
**LEA/2-AG**	*F*_(1,109)_ = 0.47, *p* = 0.50	*F*_(1,72)_ = 0.02, *p* = 0.90	*F*_(1,109)_ = 1.18, *p* = 0.28	*F*_(2,72)_ = 1.76, *p* = 0.18	***F*_(3,72)_ = 41.61, *p* < 10^−4^**			*F*_(1,72)_ = 0.15, *p* = 0.70	*F*_(1,72)_ = 0.03, *p* = 0.86
					**t_(72)_** **= 4.38,** ***p* < 10^−4^**	t_(72)_ = 2.08, *p* = 0.04	**t_(72)_** **= −4.87,** ***p* < 10^−4^**		
**OEA/2-AG**	*F*_(1,109)_ = 29.18, *p* < 10^−4^	*F*_(1,72)_ = 0.79, *p* = 0.38	*F*_(1,109)_ = 0.10, *p* = 0.75	*F*_(2,72)_ = 1.10, *p* = 0.34	***F*_(3,72)_ = 23.56, *p* < 10^−4^**			*F*_(1,72)_ = 2.10, *p* = 0.15	*F*_(1,72)_ = 0.10, *p* = 0.75
					**t_(72)_** **= 5.24,** ***p* < 10^−4^**	t_(72)_ = 1.29, *p* = 0.20	t_(72)_ = −2.52, *p* = 0.01		
**PEA/2-AG**	*F*_(1,110)_ = 84.69, *p* < 10^−4^	*F*_(1,71)_ = 0.39, *p* = 0.54	*F*_(1,110)_ = 0.23, *p* = 0.63	*F*_(2,71)_ = 1.58, *p* = 0.21	***F*_(3,71)_ = 18.41, *p* < 10^−4^**			*F*_(1,71)_ = 1.89, *p* = 0.17	*F*_(1,71)_ = 0.47, *p* = 0.50
					**t_(71)_** **= 4.95,** ***p* < 10^−4^**	t_(71)_ = 1.72, *p* = 0.09	t_(71)_ = −2.72, *p* = 0.008		
**LEA/AEA**	*F*_(1,109)_ = 102, *p* < 10^−4^	*F*_(1,72)_ = 1.08, *p* = 0.30	*F*_(1,109)_ = 1.51, *p* = 0.22	*F*_(2,72)_ = 0.58, *p* = 0.56	***F*_(3,72)_ = 17.97, *p* < 10^−4^**			*F*_(1,72)_ = 6.11, *p* = 0.02	*F*_(1,72)_ = 0.10, *p* = 0.76
					t_(72)_ = 0.08, *p* = 0.94	t_(72)_ = 1.82, *p* = 0.07	**t_(72)_** **= −4.65,** ***p* < 10^−4^**		
OEA/AEA	*F*_(1,109)_ = 280, *p* < 10^−4^	*F*_(1,72)_ = 0.08, *p* = 0.77	*F*_(1,109)_ = 0.05, *p* = 0.82	*F*_(2,72)_ = 0.97, *p* = 0.38	*F*_(3,72)_ = 1.63, *p* = 0.19			*F*_(1,72)_ = 2.18, *p* = 0.14	*F*_(1,72)_ = 0.35, *p* = 0.55
					t_(72)_ = 0.29, *p* = 0.78	t_(72)_ = 0.54, *p* = 0.59	t_(72)_ = −1.43, *p* = 0.16		
PEA/AEA	*F*_(1,110)_ = 296, *p* < 10^−4^	*F*_(1,71)_ = 0.77, *p* = 0.38	*F*_(1,110)_ = 0.18, *p* = 0.67	*F*_(2,71)_ = 0.72, *p* = 0.49	*F*_(3,71)_ = 0.72, *p* = 0.15			*F*_(1,71)_ = 2.77, *p* = 0.10	*F*_(1,71)_ = 0.50, *p* = 0.48
					t_(71)_ = −0.89, *p* = 0.38	t_(71)_ = 0.69, *p* = 0.49	t_(71)_ = −0.76, *p* = 0.45		
**Body mass index (BMI)**	*F*_(1,110)_ = 1798, *p* < 10^−4^	*F*_(1,77)_ = 2.84, *p* = 0.10	*F*_(1,110)_ = 0.41, *p* = 0.52	*F*_(2,77)_ = 0.40, *p* = 0.68	***F*_(3,77)_ = 26.88, *p* < 10^−4^**			*F*_(1,77)_ = 1.29, *p* = 0.26	*F*_(1,77)_ = 3.90, *p* = 0.05
					t_(77)_ = −0.13, *p* = 0.90	t_(77)_ = 3.59, *p* = 6 × 10^−4^	**t_(77)_** **= 4.63,** ***p* < 10^−4^**		

Significant differences (*F*-value > |8| and t-value > |4|) in the BMI, and biomolecule levels or ratios of biomolecules over time between and within the groups are marked in bold.

## Data Availability

The data that support the findings of this study are available on request from the corresponding author.

## Supplementary Materials

The following are available online at https://www.mdpi.com/article/10.3390/biomedicines10020243/s1, Table S1: Serum levels of endocannabinoids, endocannabinoid-like compounds, lipid biomolecules, and body mass index for the control subjects, first-episode psychosis (FEP) patients at baseline, after 0.6-year treatment, and in a 5.1-year follow-up treatment with antipsychotics. Table S2: Estimated effects of the complex set of predictor variables on serum concentrations of phosphatidylcholines between the control subjects, first-episode psychosis patients at baseline, after 0.6-year treatment, and after 5.1-year treatment with antipsychotics (results from the linear mixed-effects model). Figure S1: Boxplots of the variation of the prediction errors of log-transformed levels of ECs, eCB-like compounds, their ratios and BMI (derived by regressing out covariate effects) for control subjects and first episode patients at baseline, after 0.6-year, and after 5.1-year treatment with AP.
