# Supplementary material for: The Expanded Endocannabinoid System Contributes to Metabolic and Body Mass Shifts in First-Episode Schizophrenia: A 5-Year Follow-Up Study
Source: Biomedicines. 2022 Jan 24;10(2):243. doi: 10.3390/biomedicines10020243 (PMC8869544; doi:10.3390/biomedicines10020243)
Supplement: Supplementary file 1 [file biomedicines-10-00243-s001.zip › biomedicines-1517760-supplementary-.pdf]

biomedicines

MDPI

Article

The Expanded Endocannabinoid System Contributes to Metabolic and Body Mass Shifts in First-Episode Schizophrenia: A 5-Year Follow-Up Study

Madis Parksepp [1,2], Liina Haring [1,3,4,*], Kalle Kilk [4], Kadri Koch [3], Kärt Uppin [3], Raul Kangro [5], Mihkel Zilmer [4] and Eero Vasar [4]

1 Institute of Clinical Medicine, University of Tartu, 50406 Tartu, Estonia; Madis.Parksepp@vmh.ee
2 Psychiatry Clinic of Viljandi Hospital, 71024 Viljandi, Estonia
3 Psychiatry Clinic of Tartu University Hospital, 50406 Tartu, Estonia; Kadri.Koch@kliinikum.ee (K.K.); Kart.Uppin@kliinikum.ee (K.U.)
4 Centre of Excellence for Genomics and Translational Medicine, Institute of Biomedicine and Translational Medicine, Univesignallingrsity of Tartu, 50090 Tartu, Estonia; Kalle.Kilk@ut.ee (K.K.); Mihkel.Zilmer@ut.ee (M.Z.); Eero.Vasar@ut.ee (E.V.)
5 Institute of Mathematics and Statistics, University of Tartu, 50090 Tartu, Estonia; Raul.Kangro@ut.ee
* Correspondence: Liina.Haring@kliinikum.ee; Tel.: +372-7318-767

We used a linear mixed-effects modelling approach to determine the effects of the disease and antipsychotic (AP) treatment on the metabolic status of patients after the first episode of psychosis (FEP) using serum concentrations of biomolecules from the extended endocannabinoid system: endocannabinoids (eCBs): 2-arachidonoylglycerol (2AG), anandamide (AEA); endocannabinoid-like (eCB-like) N-acylethanolamines (NAEs): linoleoylethanolamide (LEA), oleoylethanolamide (OEA), palmitoylethanolamide (PEA), their potential fatty acid precursors (38 phosphatidylcholines, PCs: PC aa C24:0-PC aa C42:6) and calculated body mass index (BMI).

The AP-naïve patients' data were compared to control subjects (CSs) after adjusting for covariates (age, gender, smoking status and the time difference between visits). For the primary analysis, a set of two LME models was tested; both models use all the available data, but patient-specific determinants were only taken into account in the unrestricted models.

Regression equations for estimating body mass index (BMI), biomolecule level and their ratios	
Reduced model	fixed effects: y[log(biomolecule or BMI)]~Age+Gender+Smoking+(1∣Patient)
Unrestricted model	fixed effects: y[log(biomolecule or BMI)]~Age+Gender+Smoking+Visit+TimeDiff1+TimeDiff2+(1∣Patient)

Visit = BMI or biomolecule measurements at three time points in patients' group, Age, Gender, Smoking, Visit, TimeDiff1 is nonzero only in the case of the second visit and is equal to the difference of the actual time from the first visit and standard time (0.6 years after the first visit) of the first visit, TimeDiff2 is a similar variable which is nonzero only for the second visit, (1∣Patient) = random effects of patients.

Detailed results comparing the goodness of the two models are presented in Table S1.

According to FDR adjusted p-value (p≤0.005) derived from ANOVA comparisons, unrestricted models provided a significantly better fit than the reduced model for the change in BMI and in the levels of 33 out of 43 biomolecules over time, indicating significant differences between the levels of majority measured metabolites (77%) between the groups and within the patients' group. In addition to differences in single metabolite concentrations, we also analyzed ratios of eCBs and eCB-like NAEs (AEA/2-AG, LEA/2-AG, OEA/2-AG, PEA/2-AG, LEA/AEA, OEA/AEA, and PEA/AEA). Our analyses revealed that 71% (5 out of 7) were significantly altered in the unrestricted model in the patients' group compared to CSs. These results justified conducting of further LME regression models involving selected covariates to test the objectives of the study.

Table S1. Serum mean levels, standard deviations (SD), median and range of endocannabinoids, endocannabinoid-like compounds, lipid biomolecules (pg/ml) and body mass index for the control subjects (CSs, n=58), first-episode psychosis (FEP) patients at baseline (before treatment with antipsychotics, $FEP_{(b),}$ n=54), after 0.6-year treatment ($FEP_{(0.6\text{-year}),}$ n=47), and in a 5.1-year follow-up treatment ($FEP_{(5.1\text{-year}),}$ n=38) with antipsychotics. In a comparison between reduced and unrestricted models, p-values have been multiple tests corrected according to the false discovery rate (FRD) method. The unrestricted regression model is designated as True (i.e., more complex set of predictor variables explained more effectively biomarker level alterations over time) or False (i.e. the unrestricted model did not provide more explanatory power than the simple one).

	**CSs** mean ± SD median (range)	**FEP_b** mean ± SD median (range)	**$FEP_{(0.6\text{-year})}$** mean ± SD median (range)	**$FEP_{(5.1\text{-year})}$** mean ± SD median (range)	**Comparison between models**	
					**adjusted *p*-value**	**True (T)** or False (**F**)
**Endocannabinoids, endocannabinoid-like compounds and their ratios**						
2-Arachidonoylglycerol (2-AG) (C20:4)	3.68±1.07<br>3.60 (1.63–6.94)	3.10±1.11<br>2.94 (1.49–8.75)	3.70±1.56<br>3.45 (1.47–9.01)	6.01±2.84<br>5.37 (2.67–18.8)	$\boldsymbol{p < 10^{-12}}$	**T**
Anandamide (AEA) (C20:4, N-arachidonoyl ethanolamine)	0.24±0.15<br>0.20 (0.044–0.72)	0.35±0.24<br>0.29 (0.067–1.22)	0.25±0.17<br>0.19 (0.031–0.72)	0.29±0.18<br>0.27 (0.018–0.79)	$p = 0.001$	F
Linoleoylethanolamide (LEA) (C18:2, N-acylethanolamine)	4.20±2.55<br>3.52 (0.93–13.7)	6.47±4.37<br>5.38 (1.07–21.3)	5.73±4.26<br>4.83 (0.77–25.5)	2.69±1.71<br>2.02 (0.68–7.25)	$p < 10^{-7}$	**T**
Oleoylethanolamide (OEA) (C18:1, N-acylethanolamine)	14.3±5.90<br>14.0 (4.42–35.7)	22.3±13.5<br>18.6 (6.06–74.3)	16.8±10.1<br>13.9 (2.54–45.2)	14.9±9.88<br>12.0 (3.41–51.9)	$\boldsymbol{p = 4x10^{-4}}$	**T**
Palmitoylethanolamide (PEA) (C16:0, N-acylethanolamine)	27.7±11.4<br>25.3 (9.81–65.3)	37.1±17.6<br>33.8 (9.21–86.9)	32.3±16.2<br>27.5 (7.42–76.4)	29.1±14.6<br>25.4 (8.36–77.4)	$p = 0.002$	F
**Ratios of endocannabinoids and endocannabinoid-like compounds**						
AEA/2-AG	0.067±0.038<br>0.058 (0.015–0.18)	0.12±0.089<br>0.096 (0.017–0.47)	0.074±0.049<br>0.061 (0.0060–0.24)	0.052±0.029<br>0.047 (0.0057–0.11)	$\boldsymbol{p = 10^{-7}}$	**T**
LEA/2-AG	1.20±0.69<br>0.95 (0.28–2.89)	2.30±1.83<br>1.89 (0.38–10.9)	1.75±1.31<br>1.42 (0.30–6.73)	0.47±0.23<br>0.45 (0.084–0.92)	$\boldsymbol{p < 10^{-15}}$	**T**
OEA/2-AG	4.13±2.01<br>4.00 (1.43–12.6)	7.68±5.02<br>6.61 (1.80–28.6)	5.24±4.19<br>4.52 (0.59–25.6)	2.64±1.39<br>2.42 (0.54–6.58)	$\boldsymbol{p < 10^{-10}}$	**T**
PEA/2-AG	8.02±3.86	12.7±6.29	9.84±6.08	5.31±2.15	$\boldsymbol{p = 10^{-9}}$	**T**

	7.20 (3.28–23.1)	11.08 (3.01–33.5)	8.01 (2.22–33.9)	5.15 (1.05–11.5)		

Continued.

	**CSs mean ± SD median (range)**	**FEP_b mean ± SD median (range)**	**$FEP_{(0.6\text{-year})}$ mean ± SD median (range)**	**$FEP_{(5.1\text{-year})}$ mean ± SD median (range)**	**Comparison between models**	
					**adjusted *p*-value**	**True (T)** or False **(**F**)**
LEA/AEA	20.2±10.9<br>16.6 (5.52–49.4)	21.5±14.4<br>17.9 (5.19–14.4)	26.9±14.3<br>26.2 (5.93–75.6)	12.1±13.3<br>9.09 (2.65–84.0)	**$p < 10^{-9}$**	**T**
OEA/AEA	76.1±51.3<br>64.7 (27.1–252)	71.5±31.4<br>60.2 (25.4–155)	80.4±54.2<br>65.5 (30.5–363)	62.3±46.9<br>52.0 (26.7–303)	0.15	F
PEA/AEA	150±102<br>124 (41.9–461)	128±60.8<br>114 (47.8–333)	164±114<br>139 (44.6–782)	156±220<br>104 (55.7–1373)	0.13	F
**Body mass index**						
BMI	22.6±2.78<br>22.6 (16.8–28.9)	22.8±3.00<br>22.2 (18.4–30.2)	25.3±3.94<br>24.4 (18.8–34.7)	27.8±4.71<br>27.5 (18.8–43.0)	**$p < 10^{-11}$**	**T**
**Phosphatidylcholines (PC)**						
PC aa C24:0	0.61±0.31<br>0.53 (0.24–1.83)	0.70±0.37<br>0.57 (0.19–1.82)	0.69±0.79<br>0.50 (0.21–5.39)	0.49±0.22<br>0.46 (0.20–1.21)	0.04	F
PC aa C26:0	3.04±1.80<br>2.78 (0.82–8.40)	2.78±2.20<br>1.71 (0.80–10.3)	2.54±2.44<br>1.73 (0.90–13.8)	2.59±1.46<br>2.08 (1.00–7.24)	0.54	F
PC aa C28:1	3.04±0.77<br>2.91 (1.65–5.18)	2.68±0.73<br>2.71 (1.14–4.44)	2.93±0.87<br>2.91 (1.46–5.56)	3.21±1.25<br>2.78 (1.54–6.99)	0.65	F
PC aa C30:0	3.88±1.58<br>3.55 (2.02–10.7)	2.92±0.75<br>2.88 (1.27–4.95)	3.70±1.41<br>3.47 (1.58–8.46)	4.12±1.61<br>4.01 (1.62–7.99)	**$p = 3x10^{-4}$**	**T**
PC aa C30:2	0.28±0.20<br>0.23 (0.00–1.13)	0.23±0.20<br>0.22 (0.00–0.88)	0.24±0.19<br>0.26 (0.00–0.83)	0.04±0.09<br>0.00 (0.00–0.33)	**$p = 3x10^{-5}$**	**T**
PC aa C32:0	10.5±2.90	10.1±2.75	10.2±2.62	10.1±4.90	0.60	F

	10.4 (6.23–20.4)	10.1 (4.68–15.0)	10.3 (4.41–16.9)	9.81 (3.65–23.0)		

Continued.

	**CSs** **mean ± SD** **median (range)**	**FEP$_{b}$** **mean ± SD** **median (range)**	**FEP$_{(0.6\text{-year})}$** **mean ± SD** **median (range)**	**FEP$_{(5.1\text{-year})}$** **mean ± SD** **median (range)**	**Comparison between models**	
					**adjusted** ***p*-value**	**True (T)** or False (F)
PC aa C32:1	12.0±5.33 11.2 (4.36–33.0)	9.52±3.57 8.86 (4.00–22.1)	13.3±7.09 11.9 (4.51–36.3)	14.7±9.98 14.1 (3.15–56.7)	0.01	F
PC aa C32:2	3.37±1.62 3.16 (1.29–10.0)	1.69±0.80 1.64 (0.19–3.77)	2.82±1.31 2.90 (0.18–7.37)	2.66±1.69 2.51 (0.42–5.80)	$\boldsymbol{p = 4\text{x}10^{-9}}$	**T**
PC aa C32:3	0.48±0.18 0.45 (0.23–1.30)	0.43±0.13 0.42 (0.14–0.65)	0.47±0.15 0.47 (0.16–0.86)	0.37±0.19 0.32 (0.13–0.84)	0.005	F
PC aa C34:1	169±35.4 161 (104–256)	153±34.1 147 (87.8–230)	168±46.3 160 (87.6–291)	191±91.4 193 (73.1–423)	0.30	F
PC aa C34:2	315±72.3 301 (187–473)	241±50.0 244 (136–350)	289±73.0 287 (105–449)	345±151 303 (153–668)	$\boldsymbol{p = 2\text{x}10^{-6}}$	**T**
PC aa C34:3	12.8±4.32 12.6 (3.79–26.0)	8.04±2.53 7.75 (3.15–16.7)	12.5±5.91 11.4 (4.86–39.6)	12.5±6.45 11.1 (4.05–27.3)	$\boldsymbol{p = 2\text{x}10^{-9}}$	**T**
PC aa C34:4	1.22±0.55 1.09 (0.43–2.72)	0.60±0.26 0.57 (0.15–1.31)	1.03±0.54 0.94 (0.22–2.72)	1.27±0.67 1.21 (0.37–2.86)	$\boldsymbol{p < 10^{-12}}$	**T**
PC aa C36:0	3.03±1.84 2.70 (0.89–9.95)	3.64±1.24 3.28 (1.63–6.77)	3.59±1.25 3.30 (1.95–8.02)	1.89±0.57 1.71 (0.94–3.85)	$\boldsymbol{p < 10^{-10}}$	**T**
PC aa C36:1	37.8±9.13 37.1 (23.5–59.4)	29.3±8.73 27.7 (14.8–49.8)	40.0±14.5 37.6 (18.9–82.2)	41.2±19.2 39.4 (13.8–84.6)	$\boldsymbol{p = 3\text{x}10^{-6}}$	**T**
PC aa C36:2	191±40.8 187 (89.1–285)	132±34.7 129 (54.3–209)	187±52.7 182 (80.2–348)	214±85.0 200 (84.4–427)	$\boldsymbol{p < 10^{-12}}$	**T**
PC aa C36:3	97.0±25.5	60.1±19.2	88.5±28.1	109±42.4	$\boldsymbol{p < 10^{-13}}$	**T**

	91.2 (45.1–164)	58.2 (24.8–97.5)	85.9 (42.7–165)	98.5 (38.3–202)		
PC aa C36:4	117±36.5<br>115 (50.8–217)	90.2±33.3<br>84.7 (32.9–175)	103±40.1<br>102 (31.6–230)	153±68.2<br>130 (52.7–336)	**$p = 5 \times 10^{-7}$**	**T**

Continued.

	**CSs mean ± SD median (range)**	**FEP_b mean ± SD median (range)**	**$FEP_{(0.6\text{-year})}$ mean ± SD median (range)**	**$FEP_{(5.1\text{-year})}$ mean ± SD median (range)**	**Comparison between models**	
					**adjusted *p*-value**	**True (T)** or False **(F)**
PC aa C36:5	14.9±6.93<br>13.3 (5.56–31.9)	10.8±6.70<br>9.19 (2.45–37.7)	16.4±11.0<br>13.8 (3.74–62.7)	23.2±11.9<br>19.3 (9.52–54.8)	**$p < 10^{-9}$**	**T**
PC aa C36:6	0.76±0.29<br>0.72 (0.22–1.53)	0.54±0.21<br>0.52 (0.20–1.22)	0.77±0.34<br>0.70 (0.16–1.91)	0.76±0.35<br>0.67 (0.24–1.80)	**$p = 4 \times 10^{-5}$**	**T**
PC aa C38:0	2.62±1.08<br>2.43 (1.01–6.66)	2.56±0.66<br>2.54 (1.32–4.41)	2.77±0.89<br>2.71 (1.17–6.85)	2.19±0.65<br>2.03 (1.27–4.35)	0.007	F
PC aa C38:1	1.90±1.27<br>1.44 (0.08–5.25)	2.32±1.17<br>2.11 (0.89–6.84)	2.38±1.20<br>2.15 (0.68–6.25)	0.95±0.38<br>0.89 (0.42–2.15)	**$p < 10^{-10}$**	**T**
PC aa C38:3	30.9±6.65<br>30.5 (19.0–47.6)	23.5±6.05<br>22.6 (11.7–41.6)	31.6±11.2<br>30.1 (13.7–64.8)	39.5±17.2<br>37.5 (14.1–79.9)	**$p < 10^{-9}$**	**T**
PC aa C38:4	59.0±16.5<br>58.3 (29.2–103)	46.2±19.0<br>44.7 (20.0–114)	54.4±22.6<br>54.1 (21.6–136)	83.7±37.1<br>73.1 (29.2–178)	**$p < 10^{-9}$**	**T**
PC aa C38:5	29.3±7.94<br>29.5 (13.3–47.7)	22.5±9.50<br>21.4 (8.08–50.9)	29.5±14.3<br>26.8 (9.27–79.3)	45.6±18.7<br>38.0 (20.9–87.8)	**$p < 10^{-12}$**	**T**
PC aa C38:6	48.5±24.5<br>42.7 (15.5–138)	36.2±18.5<br>30.2 (10.0–94.0)	41.3±20.4<br>38.8 (9.81–96.0)	67.2±24.1<br>61.2 (29.0–113)	**$p < 10^{-9}$**	**T**
PC aa C40:1	0.74±0.55<br>0.63 (0.000–2.08)	1.06±0.35<br>1.01 (0.42–2.27)	1.01±0.43<br>0.94 (0.45–2.42)	0.36±0.09<br>0.36 (0.000–0.50)	**$p < 10^{-11}$**	**T**
PC aa C40:2	1.56±1.24<br>1.47 (0.19–4.00)	2.58±1.02<br>2.50 (0.58–5.88)	2.14±1.03<br>2.02 (0.52–4.63)	0.33±0.11<br>0.31 (0.16–0.65)	**$p < 10^{-39}$**	**T**

PC aa C40:3	1.55±1.10<br>1.43 (0.31–3.77)	2.40±0.95<br>2.31 (0.69–5.65)	2.15±1.04<br>2.00 (0.62–4.97)	0.52±0.15<br>0.50 (0.23–0.80)	$p < 10^{-33}$	**T**
PC aa C40:4	3.65±1.56<br>3.40 (1.46–8.09)	4.38±1.27<br>4.10 (2.27–8.16)	4.30±1.32<br>3.95 (2.29–9.73)	2.79±1.16<br>2.42 (1.15–5.75)	$p < 10^{-8}$	**T**

Continued.

	**CSs mean ± SD median (range)**	**FEP_b mean ± SD median (range)**	**$FEP_{(0.6\text{-year})}$ mean ± SD median (range)**	**$FEP_{(5.1\text{-year})}$ mean ± SD median (range)**	**Comparison between models**	
					**adjusted p-value**	**True (T) or false (F)**
PC aa C40:5	5.16±1.38<br>4.79 (2.73–10.5)	4.07±1.48<br>3.88 (1.88–8.61)	5.68±2.82<br>4.98 (1.92–17.0)	7.89±3.88<br>7.12 (2.67–19.2)	$p < 10^{-10}$	**T**
PC aa C40:6	15.9±7.55<br>15.2 (5.38–49.0)	11.2±5.30<br>9.45 (3.93–26.3)	14.8±7.62<br>14.6 (3.94–42.5)	24.3±9.92<br>20.4 (10.3–50.0)	$p < 10^{-12}$	**T**
PC aa C42:0	0.61±0.27<br>0.56 (0.21–1.79)	0.75±0.20<br>0.75 (0.38–1.27)	0.69±0.22<br>0.65 (0.30–1.34)	0.44±0.13<br>0.42 (0.24–0.79)	$p < 10^{-10}$	**T**
PC aa C42:1	0.40±0.19<br>0.35 (0.14–1.05)	0.54±0.17<br>0.53 (0.27–1.00)	0.50±0.20<br>0.45 (0.22–1.04)	0.23±0.065<br>0.22 (0.14–0.40)	$p < 10^{-19}$	**T**
PC aa C42:2	0.49±0.31<br>0.44 (0.11–1.41)	0.72±0.23<br>0.67 (0.31–1.37)	0.68±0.29<br>0.64 (0.21–1.46)	0.21±0.063<br>0.21 (0.10–0.39)	$p < 10^{-33}$	**T**
PC aa C42:4	0.65±0.48<br>0.70 (0.08–1.60)	0.94±0.33<br>0.91 (0.32–2.06)	0.91±0.41<br>0.88 (0.27–2.51)	0.16±0.052<br>0.15 (0.069–0.30)	$p < 10^{-36}$	**T**
PC aa C42:5	0.51±0.25<br>0.48 (0.15–1.26)	0.57±0.15<br>0.55 (0.32–1.05)	0.59±0.18<br>0.55 (0.34–1.36)	0.33±0.14<br>0.32 (0.12–0.93)	$p < 10^{-9}$	**T**
PC aa C42:6	0.73±0.20<br>0.67 (0.46–1.61)	0.72±0.20<br>0.69 (0.35–1.28)	0.71±0.18<br>0.68 (0.40–1.16)	0.58±0.19<br>0.59 (0.27–0.97)	$p = 3 \times 10^{-5}$	**T**

Table S2. Estimated effects of the complex set of predictor variables on serum concentrations of phosphatidylcholines between the control subjects (CSs, n=58), first-episode psychosis patients at baseline (before treatment with antipsychotics, $FEP_{(b)}$, n=54), after 0.6-year treatment ($FEP_{(0.6\text{-}year)}$, n=47), and after 5.1-year treatment ($FEP_{(5.1\text{-}year)}$, n=38) with antipsychotics (results from the linear mixed-effects model).

	Intercept	Age	Gender	Smoking	**Disease and treatment effect**			TimeDiff1	TimeDiff2
					**FEP patients before treatment**	**FEP patients after 0.6-year treatment**	**FEP patients after 5.1-year treatment**		
	Effects of independent variables on the dependent variable (*F*-value, *p*-value)								
					t-value, *p*-value				
**Phosphatidylcholines (PC)**									
PC aa C24:0	$F_{(1,109)} = 19.23$,	$F_{(1,74)} = 2.01$,	$F_{(1,109)} = 1.07$,	$F_{(2,74)} = 0.74$,	$F_{(3,74)} = 3.94$, $p = 0.01$			$F_{(1,74)} = 0.06$,	$F_{(1,74)} = 0.005$,
	$p < 10^{-4}$	$p = 0.16$	$p = 0.30$	$p = 0.48$	$t_{(74)} = 0.70$, $p = 0.49$	$t_{(74)} = -0.56$, $p = 0.57$	$t_{(74)} = -2.55$, $p = 0.01$	$p = 0.80$	$p = 0.95$
PC aa C26:0	$F_{(1,109)} = 10.09$,	$F_{(1,74)} = 0.70$,	$F_{(1,109)} = 1.96$,	$F_{(2,74)} = 0.90$,	$F_{(3,74)} = 1.21$, $p = 0.31$			$F_{(1,74)} = 0.04$,	$F_{(1,74)} = 0.07$,
	$p = 0.002$	$p = 0.41$	$p = 0.16$	$p = 0.41$	$t_{(74)} = -1.01$, $p = 0.31$	$t_{(74)} = -1.85$, $p = 0.07$	$t_{(74)} = -0.45$, $p = 0.65$	$p = 0.83$	$p = 0.78$
PC aa C28:1	$F_{(1,109)} = 42.36$,	$F_{(1,74)} = 0.43$,	$F_{(1,109)} = 2.21$,	$F_{(2,74)} = 1.30$,	$F_{(3,74)} = 2.59$, $p = 0.06$			$F_{(1,74)} = 3.72$,	$F_{(1,74)} = 0.06$,
	$p < 10^{-4}$	$p = 0.51$	$p = 0.14$	$p = 0.28$	$t_{(74)} = -2.02$, $p = 0.05$	$t_{(74)} = -0.94$, $p = 0.35$	$t_{(74)} = 0.57$, $p = 0.57$	$p = 0.06$	$p = 0.80$
PC aa C30:0	$F_{(1,109)} = 38.41$,	$F_{(1,74)} = 0.53$,	$F_{(1,109)} = 3.68$,	$F_{(2,74)} = 0.29$,	$F_{(3,74)} = 7.40$, $p = 0.0002$			$F_{(1,74)} = 4.13$,	$F_{(1,74)} = 0.02$,
	$p < 10^{-4}$	$p = 0.47$	$p = 0.06$	$p = 0.75$	$t_{(74)} = -3.44$, $p = 0.001$	$t_{(74)} = -0.69$, $p = 0.49$	$t_{(74)} = 0.54$, $p = 0.59$	$p = 0.05$	$p = 0.88$
PC aa C30:2	$F_{(1,109)} = 10.80$,	$F_{(1,74)} = 0.25$,	$F_{(1,109)} = 1.02$,	$F_{(2,74)} = 0.31$,	**$F_{(3,74)} = 9.27$, $p < 10^{-4}$**			$F_{(1,74)} = 1.20$,	$F_{(1,74)} = 0.05$,
	$p = 0.001$	$p = 0.62$	$p = 0.31$	$p = 0.73$	$t_{(74)} = -1.30$, $p = 0.20$	$t_{(74)} = -1.33$, $p = 0.19$	**$t_{(74)} = -5.11$, $p < 10^{-4}$**	$p = 0.28$	$p = 0.83$
PC aa C32:0	$F_{(1,109)} = 168$,	$F_{(1,74)} = 1.73$,	$F_{(1,109)} = 0.33$,	$F_{(2,74)} = 0.91$,	$F_{(3,74)} = 1.05$, $p = 0.38$			$F_{(1,74)} = 0.06$,	$F_{(1,74)} = 0.31$,
	$p < 10^{-4}$	$p = 0.19$	$p = 0.57$	$p = 0.41$	$t_{(74)} = -0.72$, $p = 0.47$	$t_{(74)} = -1.03$, $p = 0.30$	$t_{(74)} = -1.70$, $p = 0.09$	$p = 0.81$	$p = 0.58$
PC aa C32:1	$F_{(1,109)} = 88.19$,	$F_{(1,74)} = 0.03$,	$F_{(1,109)} = 1.08$,	$F_{(2,74)} = 0.47$,	$F_{(3,74)} = 4.78$, $p = 0.004$			$F_{(1,74)} = 2.39$,	$F_{(1,74)} = 0.13$,
	$p < 10^{-4}$	$p = 0.86$	$p = 0.30$	$p = 0.62$	$t_{(74)} = -2.04$, $p = 0.04$	$t_{(74)} = 0.33$, $p = 0.74$	$t_{(74)} = 0.56$, $p = 0.58$	$p = 0.13$	$p = 0.72$
PC aa C32:2	$F_{(1,109)} = 10.51$,	$F_{(1,74)} = 0.12$,	$F_{(1,109)} = 1.72$,	$F_{(2,74)} = 0.36$,	**$F_{(3,74)} = 17.59$, $p < 10^{-4}$**			$F_{(1,74)} = 4.50$,	$F_{(1,74)} = 0.56$,
	$p = 0.002$	$p = 0.73$	$p = 0.19$	$p = 0.70$	**$t_{(74)} = -5.92$, $p < 10^{-4}$**	$t_{(74)} = -2.06$, $p = 0.04$	$t_{(74)} = -2.43$, $p = 0.02$	$p = 0.04$	$p = 0.46$

Continued.

	Intercept	Age	Gender	Smoking	**Disease and treatment effect**			TimeDiff1	TimeDiff2
					**FEP patients before treatment**	**FEP patients after 0.6-year treatment**	**FEP patients after 5.1-year treatment**		
	Effects of independent variables on the dependent variable (F-value, p-value)								
					t-value, p-value				
PC aa C32:3	$F_{(1,109)} = 39.11$, $p < 10^{-4}$	$F_{(1,74)} = 1.59$, $p = 0.21$	$F_{(1,109)} = 3.95$, $p = 0.05$	$F_{(2,74)} = 1.02$, $p = 0.36$	$F_{(3,74)} = 5.07$, $p = 0.003$			$F_{(3,74)} = 5.07$, $p = 0.003$	$F_{(3,74)} = 5.07$, $p = 0.003$
					$t_{(74)} = -1.15$, $p = 0.25$	$t_{(74)} = -1.15$, $p = 0.25$	$t_{(74)} = -1.15$, $p = 0.25$		
PC aa C34:1	$F_{(1,109)} = 1107$, $p < 10^{-4}$	$F_{(1,74)} = 0.48$, $p = 0.49$	$F_{(1,109)} = 0.42$, $p = 0.52$	$F_{(2,74)} = 0.14$, $p = 0.87$	$F_{(3,74)} = 1.91$, $p = 0.13$			$F_{(3,74)} = 1.91$, $p = 0.13$	$F_{(3,74)} = 1.91$, $p = 0.13$
					$t_{(74)} = -1.67$, $p = 0.10$	$t_{(74)} = -1.67$, $p = 0.10$	$t_{(74)} = -1.67$, $p = 0.10$		
PC aa C34:2	$F_{(1,109)} = 1668$, $p < 10^{-4}$	$F_{(1,74)} = 0.19$, $p = 0.66$	$F_{(1,109)} = 1.20$, $p = 0.27$	$F_{(2,74)} = 0.13$, $p = 0.88$	**$F_{(3,74)} = 11.47$, $p < 10^{-4}$**			$F_{(3,74)} = 11.47$, $p < 10^{-4}$	$F_{(3,74)} = 11.47$, $p < 10^{-4}$
					**$t_{(74)} = -4.27$, $p = 10^{-4}$**	$t_{(74)} = -1.60$, $p = 0.11$	$t_{(74)} = 0.67$, $p = 0.51$		
PC aa C34:3	$F_{(1,109)} = 169$, $p < 10^{-4}$	$F_{(1,74)} = 0.39$, $p = 0.54$	$F_{(1,109)} = 1.65$, $p = 0.20$	$F_{(2,74)} = 0.10$, $p = 0.91$	**$F_{(3,74)} = 20.19$, $p < 10^{-4}$**			$F_{(1,74)} = 1.79$, $p = 0.18$	$F_{(1,74)} = 1.45$, $p = 0.23$
					**$t_{(74)} = -5.54$, $p < 10^{-4}$**	$t_{(74)} = -0.66$, $p = 0.51$	$t_{(74)} = -0.70$, $p = 0.49$		
PC aa C34:4	$F_{(1,109)} = 0.02$, $p = 0.88$	$F_{(1,74)} = 0.15$, $p = 0.70$	$F_{(1,109)} = 1.01$, $p = 0.32$	$F_{(2,74)} = 0.05$, $p = 0.95$	**$F_{(3,74)} = 30.09$, $p < 10^{-4}$**			$F_{(1,74)} = 1.74$, $p = 0.19$	$F_{(1,74)} = 0.70$, $p = 0.40$
					**$t_{(74)} = -7.05$, $p < 10^{-4}$**	$t_{(74)} = -2.17$, $p = 0.03$	$t_{(74)} = 0.04$, $p = 0.97$		
PC aa C36:0	$F_{(1,109)} = 1.85$, $p = 0.18$	$F_{(1,74)} = 10.53$, $p = 0.002$	$F_{(1,109)} = 2.97$, $p = 0.09$	$F_{(2,74)} = 0.34$, $p = 0.71$	**$F_{(3,74)} = 21.46$, $p < 10^{-4}$**			$F_{(1,74)} = 1.56$, $p = 0.21$	$F_{(1,74)} = 0.16$, $p = 0.69$
					$t_{(74)} = 3.52$, $p = 7 \times 10^{-4}$	$t_{(74)} = 3.23$, $p = 0.002$	**$t_{(74)} = -4.40$, $p < 10^{-4}$**		
PC aa C36:1	$F_{(1,109)} = 471$, $p < 10^{-4}$	$F_{(1,74)} = 1.22$, $p = 0.27$	$F_{(1,109)} = 0.20$, $p = 0.66$	$F_{(2,74)} = 0.64$, $p = 0.53$	**$F_{(3,74)} = 12.33$, $p < 10^{-4}$**			$F_{(1,74)} = 1.40$, $p = 0.24$	$F_{(1,74)} = 1.48$, $p = 0.23$
					**$t_{(74)} = -4.12$, $p = 10^{-4}$**	$t_{(74)} = 0.14$, $p = 0.89$	$t_{(74)} = -0.50$, $p = 0.61$		
PC aa C36:2	$F_{(1,109)} = 1600$, $p < 10^{-4}$	$F_{(1,74)} = 0.10$, $p = 0.75$	$F_{(1,109)} = 0.35$, $p = 0.55$	$F_{(2,74)} = 0.42$, $p = 0.66$	**$F_{(3,74)} = 26.21$, $p < 10^{-4}$**			$F_{(1,74)} = 2.68$, $p = 0.11$	$F_{(1,74)} = 5.69$, $p = 0.02$
					**$t_{(74)} = -6.83$, $p < 10^{-4}$**	$t_{(74)} = -0.77$, $p = 0.44$	$t_{(74)} = 0.70$, $p = 0.48$		
PC aa C36:3	$F_{(1,109)} = 941$, $p < 10^{-4}$	$F_{(1,74)} = 0.64$, $p = 0.43$	$F_{(1,109)} = 0.21$, $p = 0.64$	$F_{(2,74)} = 1.07$, $p = 0.35$	**$F_{(3,74)} = 31.10$, $p < 10^{-4}$**			$F_{(1,74)} = 0.92$, $p = 0.34$	$F_{(1,74)} = 0.47$, $p = 0.49$
					**$t_{(74)} = -7.64$, $p < 10^{-4}$**	$t_{(74)} = -1.54$, $p = 0.13$	$t_{(74)} = 0.94$, $p = 0.35$		
PC aa C36:4	$F_{(1,109)} = 666$, $p < 10^{-4}$	$F_{(1,74)} = 0.004$, $p = 0.95$	$F_{(1,109)} = 1.10$, $p = 0.30$	$F_{(2,74)}=1.08$, $p = 0.35$	**$F_{(3,74)} = 13.72$, $p < 10^{-4}$**			$F_{(1,74)} = 0.10$, $p = 0.75$	$F_{(1,74)} = 0.45$, $p = 0.51$
					$t_{(74)}=-3.78$, $p = 3 \times 10^{-4}$	$t_{(74)} = -2.21$, $p = 0.03$	$t_{(74)} = 2.65$, $p = 0.01$		

Continued.

	Intercept	Age	Gender	Smoking	**Disease and treatment effect**			TimeDiff1	TimeDiff2
					**FEP patients before treatment**	**FEP patients after 0.6-year treatment**	**FEP patients after 5.1-year treatment**		
	Effects of independent variables on the dependent variable (*F*-value, *p*-value)								
					t-value, *p*-value				
PC aa C36:5	$F_{(1,108)} = 89.82$, $p < 10^{-4}$	$F_{(1,74)} = 0.25$, $p = 0.62$	$F_{(1,108)} = 0.65$, $p = 0.42$	$F_{(2,74)} = 2.11$, $p = 0.13$	**$F_{(3,74)} = 19.22$, $p < 10^{-4}$**			$F_{(1,74)} = 0.78$, $p = 0.38$	$F_{(1,74)} = 0.84$, $p = 0.36$
					$t_{(74)} = -3.56$, $p = 7\text{x}10^{-4}$	$t_{(74)} = 0.02$, $p = 0.99$	$t_{(74)} = 3.33$, $p = 0.001$		
PC aa C36:6	$F_{(1,109)} = 11.65$, $p = 9\text{x}10^{-4}$	$F_{(1,74)} = 0.22$, $p = 0.64$	$F_{(1,109)} = 5.81$, $p = 0.02$	$F_{(2,74)} = 0.67$, $p = 0.51$	**$F_{(3,74)} = 9.64$, $p < 10^{-4}$**			$F_{(1,74)} = 0.83$, $p = 0.37$	$F_{(1,74)} = 1.12$, $p = 0.29$
					$t_{(74)} = -3.60$, $p = 6\text{x}10^{-4}$	$t_{(74)} = -0.17$, $p = 0.86$	$t_{(74)} = -0.11$, $p = 0.91$		
PC aa C38:0	$F_{(1,109)} = 6.62$, $p = 0.01$	$F_{(1,74)} = 4.64$, $p = 0.03$	$F_{(1,109)} = 11.71$, $p = 9\text{x}10^{-4}$	$F_{(2,74)} = 0.58$, $p = 0.56$	$F_{(3,74)} = 5.65$, $p = 0.001$			$F_{(1,74)} = 0.13$, $p = 0.72$	$F_{(1,74)} = 0.21$, $p = 0.64$
					$t_{(74)} = 0.63$, $p = 0.53$	$t_{(74)} = 1.85$, $p = 0.07$	$t_{(74)} = -2.19$, $p = 0.03$		
PC aa C38:1	$F_{(1,109)} = 0.54$, $p = 0.46$	$F_{(1,74)} = 3.81$, $p = 0.05$	$F_{(1,109)} = 1.09$, $p = 0.30$	$F_{(2,74)} = 0.97$, $p = 0.38$	**$F_{(3,74)} = 23.54$, $p < 10^{-4}$**			$F_{(1,74)} = 6.98$, $p = 0.01$	$F_{(1,74)} = 1.39$, $p = 0.24$
					$t_{(74)} = 2.91$, $p = 0.005$	$t_{(74)} = 2.72$, $p = 0.008$	**$t_{(74)} = 4.47$, $p < 10^{-4}$**		
PC aa C38:3	$F_{(1,109)} = 564$, $p < 10^{-4}$	$F_{(1,74)} = 0.15$, $p = 0.69$	$F_{(1,109)} = 0.18$, $p = 0.67$	$F_{(2,74)} = 0.28$, $p = 0.75$	**$F_{(3,74)} = 19.21$, $p < 10^{-4}$**			$F_{(1,74)} = 0.46$, $p = 0.50$	$F_{(1,74)} = 1.59$, $p = 0.21$
					**$t_{(74)} = -4.67$, $p < 10^{-4}$**	$t_{(74)} = -0.25$, $p = 0.80$	$t_{(74)} = 2.34$, $p = 0.02$		
PC aa C38:4	$F_{(1,109)} = 559$, $p < 10^{-4}$	$F_{(1,74)} = 0.32$, $p = 0.57$	$F_{(1,109)} = 0.47$, $p = 0.49$	$F_{(2,74)} = 2.18$, $p = 0.12$	**$F_{(3,74)} = 17.70$, $p < 10^{-4}$**			$F_{(1,74)} = 0.32$, $p = 0.57$	$F_{(1,74)} = 1.68$, $p = 0.20$
					$t_{(74)} = -3.78$, $p = 3\text{x}10^{-4}$	$t_{(74)} = -1.65$, $p = 0.10$	$t_{(74)} = 3.69$, $p = 4\text{x}10^{-4}$		
PC aa C38:5	$F_{(1,108)} = 365$, $p < 10^{-4}$	$F_{(1,74)} = 0.26$, $p = 0.61$	$F_{(1,108)} = 0.02$, $p = 0.89$	$F_{(2,74)} = 2.02$, $p = 0.14$	**$F_{(3,74)} = 25.75$, $p < 10^{-4}$**			$F_{(1,74)} = 0.66$, $p = 0.42$	$F_{(1,74)} = 0.76$, $p = 0.39$
					**$t_{(74)} = -4.05$, $p = 10^{-4}$**	$t_{(74)} = -0.83$, $p = 0.41$	**$t_{(74)} = 4.67$, $p < 10^{-4}$**		
PC aa C38:6	$F_{(1,109)} = 283$, $p < 10^{-4}$	$F_{(1,74)} = 0.86$, $p = 0.36$	$F_{(1,109)} = 8.89$, $p = 0.004$	$F_{(2,74)} = 5.79$, $p = 0.005$	**$F_{(3,74)} = 18.10$, $p < 10^{-4}$**			$F_{(1,74)} = 2.92$, $p = 0.09$	$F_{(1,74)} = 0.44$, $p = 0.51$
					$t_{(74)} = -2.79$, $p = 0.007$	$t_{(74)} = -1.37$, $p = 0.18$	**$t_{(74)} = 4.62$, $p < 10^{-4}$**		
PC aa C40:1	$F_{(1,109)} = 0.86$, $p = 0.36$	$F_{(1,74)} = 5.91$, $p = 0.02$	$F_{(1,109)} = 1.66$, $p = 0.20$	$F_{(2,74)} = 0.33$, $p = 0.72$	**$F_{(3,74)} = 33.48$, $p < 10^{-4}$**			$F_{(1,74)} = 0.34$, $p = 0.56$	$F_{(1,74)} = 0.24$, $p = 0.63$
					$t_{(74)} = 3.86$, $p = 2\text{x}10^{-4}$	$t_{(74)} = 3.18$, $p = 0.002$	**$t_{(74)} = -4.76$, $p < 10^{-4}$**		
PC aa C40:2	$F_{(1,109)} = 6.72$,	$F_{(1,74)} = 7.89$,	$F_{(1,109)} = 0.35$,	$F_{(2,74)} = 0.09$,	**$F_{(3,74)} = 182$, $p < 10^{-4}$**			$F_{(1,74)} = 0.34$,	$F_{(1,74)} = 0.24$,

	$p = 0.01$	$p = 0.006$	$p = 0.56$	$p = 0.92$	**$t_{(74)} = 5.29$, $p < 10^{-4}$**	$t_{(74)} = 3.65$, $p = 5 \times 10^{-4}$	**$t_{(74)} = -7.51$, $p < 10^{-4}$**	$p = 0.56$	$p = 0.63$

Continued.

	Intercept	Age	Gender	Smoking	**Disease and treatment effect**			TimeDiff1	TimeDiff2
					**FEP patients before treatment**	**FEP patients after 0.6-year treatment**	**FEP patients after 5.1-year treatment**		
	Effects of independent variables on the dependent variable (*F*-value, *p*-value)								
					t-value, *p*-value				
PC aa C40:3	$F_{(1,109)} = 7.90$, $p = 0.006$	$F_{(1,74)} = 11.29$, $p = 0.001$	$F_{(1,109)} = 0.66$, $p = 0.42$	$F_{(2,74)} = 0.09$, $p = 0.92$	**$F_{(3,74)} = 133$, $p < 10^{-4}$**			$F_{(1,74)} = 0.51$, $p = 0.48$	$F_{(1,74)} = 5.34$, $p = 0.02$
					**$t_{(74)} = 4.94$, $p < 10^{-4}$**	$t_{(74)} = 3.62$, $p = 5 \times 10^{-4}$	**$t_{(74)} = -7.10$, $p < 10^{-4}$**		
PC aa C40:4	$F_{(1,109)} = 18.10$, $p < 10^{-4}$	$F_{(1,74)} = 14.05$, $p = 4 \times 10^{-4}$	$F_{(1,109)} = 0.02$, $p = 0.90$	$F_{(2,74)} = 0.17$, $p = 0.84$	**$F_{(3,74)} = 19.01$, $p < 10^{-4}$**			$F_{(1,74)} = 0.35$, $p = 0.56$	$F_{(1,74)} = 0.15$, $p = 0.70$
					$t_{(74)} = 2.85$, $p = 0.006$	$t_{(74)} = 2.18$, $p = 0.03$	**$t_{(74)} = -4.59$, $p < 10^{-4}$**		
PC aa C40:5	$F_{(1,109)} = 99.21$, $p < 10^{-4}$	$F_{(1,74)} = 0.06$, $p = 0.81$	$F_{(1,109)} = 0.80$, $p = 0.37$	$F_{(2,74)} = 0.84$, $p = 0.44$	**$F_{(3,74)} = 22.21$, $p < 10^{-4}$**			$F_{(1,74)} = 0.05$, $p = 0.82$	$F_{(1,74)} = 1.12$, $p = 0.29$
					$t_{(74)} = -3.70$, $p = 4 \times 10^{-4}$	$t_{(74)} = 0.18$, $p = 0.86$	$t_{(74)} = 3.85$, $p = 3 \times 10^{-4}$		
PC aa C40:6	$F_{(1,109)} = 159$, $p < 10^{-4}$	$F_{(1,74)} = 0.67$, $p = 0.41$	$F_{(1,109)} = 5.89$, $p = 0.02$	$F_{(2,74)} = 4.38$, $p = 0.02$	**$F_{(3,74)} = 25.97$, $p < 10^{-4}$**			$F_{(1,74)} = 1.50$, $p = 0.22$	$F_{(1,74)} = 1.67$, $p = 0.20$
					$t_{(74)} = -3.67$, $p = 5 \times 10^{-4}$	$t_{(74)} = -0.77$, $p = 0.44$	**$t_{(74)} = 5.21$, $p < 10^{-4}$**		
PC aa C42:0	$F_{(1,109)} = 58.60$, $p < 10^{-4}$	$F_{(1,74)} = 7.10$, $p = 0.009$	$F_{(1,109)} = 10.10$, $p = 0.002$	$F_{(2,74)} = 0.26$, $p = 0.77$	**$F_{(3,74)} = 23.17$, $p < 10^{-4}$**			$F_{(1,74)} = 0.009$, $p = 0.93$	$F_{(1,74)} = 0.04$, $p = 0.84$
					**$t_{(74)} = 4.02$, $p = 10^{-4}$**	$t_{(74)} = 2.60$, $p = 0.01$	**$t_{(74)} = -4.33$, $p < 10^{-4}$**		
PC aa C42:1	$F_{(1,109)} = 93.04$, $p < 10^{-4}$	$F_{(1,74)} = 11.53$, $p = 0.001$	$F_{(1,109)} = 5.19$, $p = 0.02$	$F_{(2,74)} = 0.32$, $p = 0.73$	**$F_{(3,74)} = 51.73$, $p < 10^{-4}$**			$F_{(1,74)} = 0.34$, $p = 0.56$	$F_{(1,74)} = 2.35$, $p = 0.13$
					**$t_{(74)} = 5.10$, $p < 10^{-4}$**	$t_{(74)} = 3.48$, $p = 9 \times 10^{-4}$	**$t_{(74)} = -6.31$, $p < 10^{-4}$**		
PC aa C42:2	$F_{(1,109)} = 57.31$, $p < 10^{-4}$	$F_{(1,74)} = 12.72$, $p = 6 \times 10^{-4}$	$F_{(1,109)} = 4.12$, $p = 0.04$	$F_{(2,74)} = 0.12$, $p = 0.88$	**$F_{(3,74)} = 131$, $p < 10^{-4}$**			$F_{(1,74)} = 0.35$, $p = 0.55$	$F_{(1,74)} = 2.58$, $p = 0.11$
					**$t_{(74)} = 5.26$, $p < 10^{-4}$**	**$t_{(74)} = 4.04$, $p = 10^{-4}$**	**$t_{(74)} = -7.40$, $p < 10^{-4}$**		
PC aa C42:4	$F_{(1,109)} = 26.80$, $p < 10^{-4}$	$F_{(1,74)} = 9.66$, $p = 0.003$	$F_{(1,109)} = 0.79$, $p = 0.37$	$F_{(2,74)} = 0.29$, $p = 0.75$	**$F_{(3,74)} = 155$, $p < 10^{-4}$**			$F_{(1,74)} = 0.39$, $p = 0.53$	$F_{(1,74)} = 4.99$, $p = 0.03$
					**$t_{(74)} = 4.57$, $p < 10^{-4}$**	$t_{(74)} = 3.71$, $p = 4 \times 10^{-4}$	**$t_{(74)} = -7.85$, $p < 10^{-4}$**		
PC aa C42:5	$F_{(1,109)} = 51.54$, $p < 10^{-4}$	$F_{(1,74)} = 5.35$, $p = 0.02$	$F_{(1,109)} = 3.65$, $p = 0.06$	$F_{(2,74)} = 0.01$, $p = 0.99$	**$F_{(3,74)} = 21.60$, $p < 10^{-4}$**			$F_{(1,74)} = 0.23$, $p = 0.63$	$F_{(1,74)} = 0.001$, $p = 0.97$
					$t_{(74)} = 2.60$, $p = 0.01$	$t_{(74)} = 2.61$, $p = 0.01$	**$t_{(74)} = -4.77$, $p < 10^{-4}$**		

**PC aa C42:6**	$F_{(1,109)} = 24.56$, $p < 10^{-4}$	$F_{(1,74)} = 4.17$, $p = 0.04$	$F_{(1,109)} = 1.54$, $p = 0.22$	$F_{(2,74)} = 0.88$, $p = 0.42$	$\mathbf{F_{(3,74)} = 9.82, p < 10^{-4}}$			$F_{(1,74)} = 1.35$, $p = 0.25$	$F_{(1,74)} = 3x10^{-4}$, $p = 0.99$
					$t_{(74)} = -0.53$, $p = 0.59$	$t_{(74)} = -0.96$, $p = 0.34$	$\mathbf{t_{(74)} = -4.69, p < 10^{-4}}$		

Significant differences (F-value >|8| and t-value > |4|) in the biomolecule levels over time between and within the groups are marked in bold.

The figures comprise only those analyzed characteristics for which the effect of treatment and the effect of disease duration F-value was > |8|, according to the linear mixed-effects models.

Phosphatidylcholines

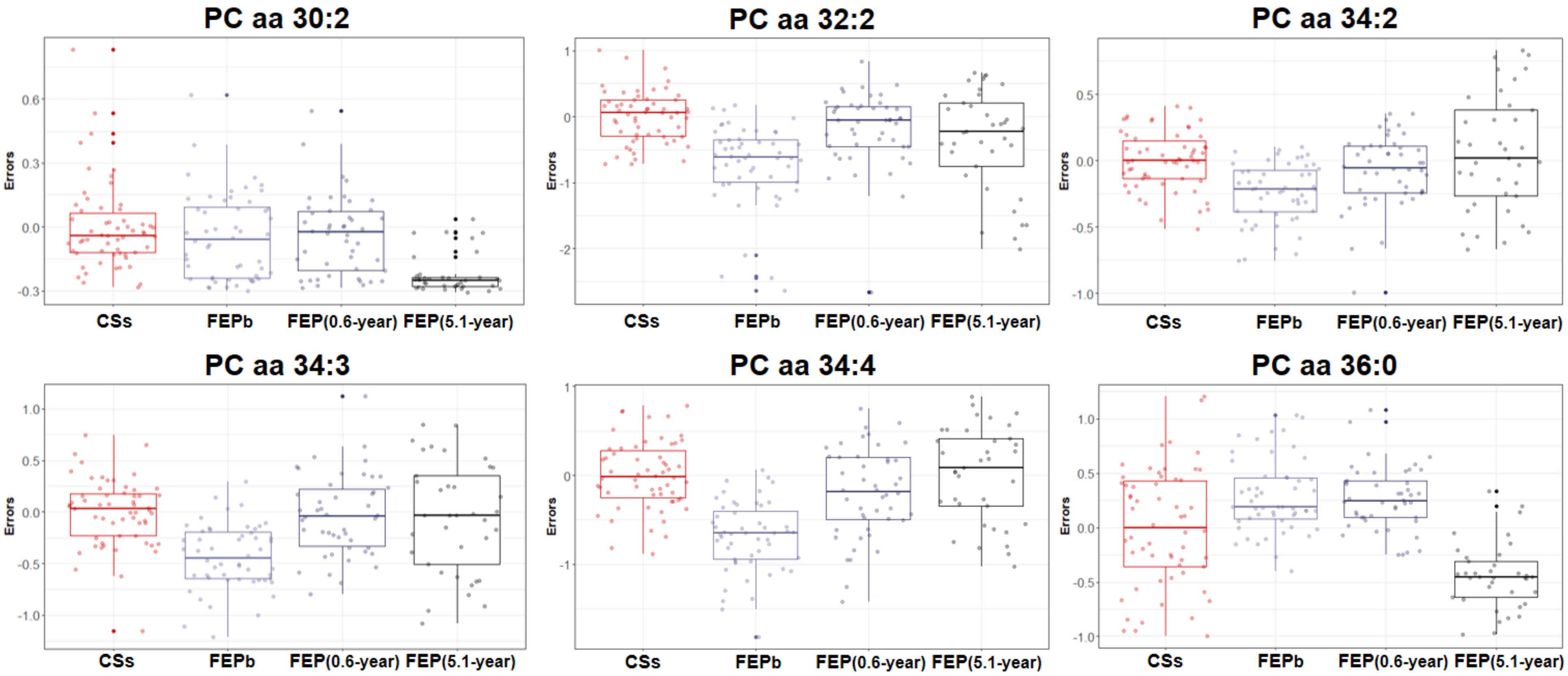

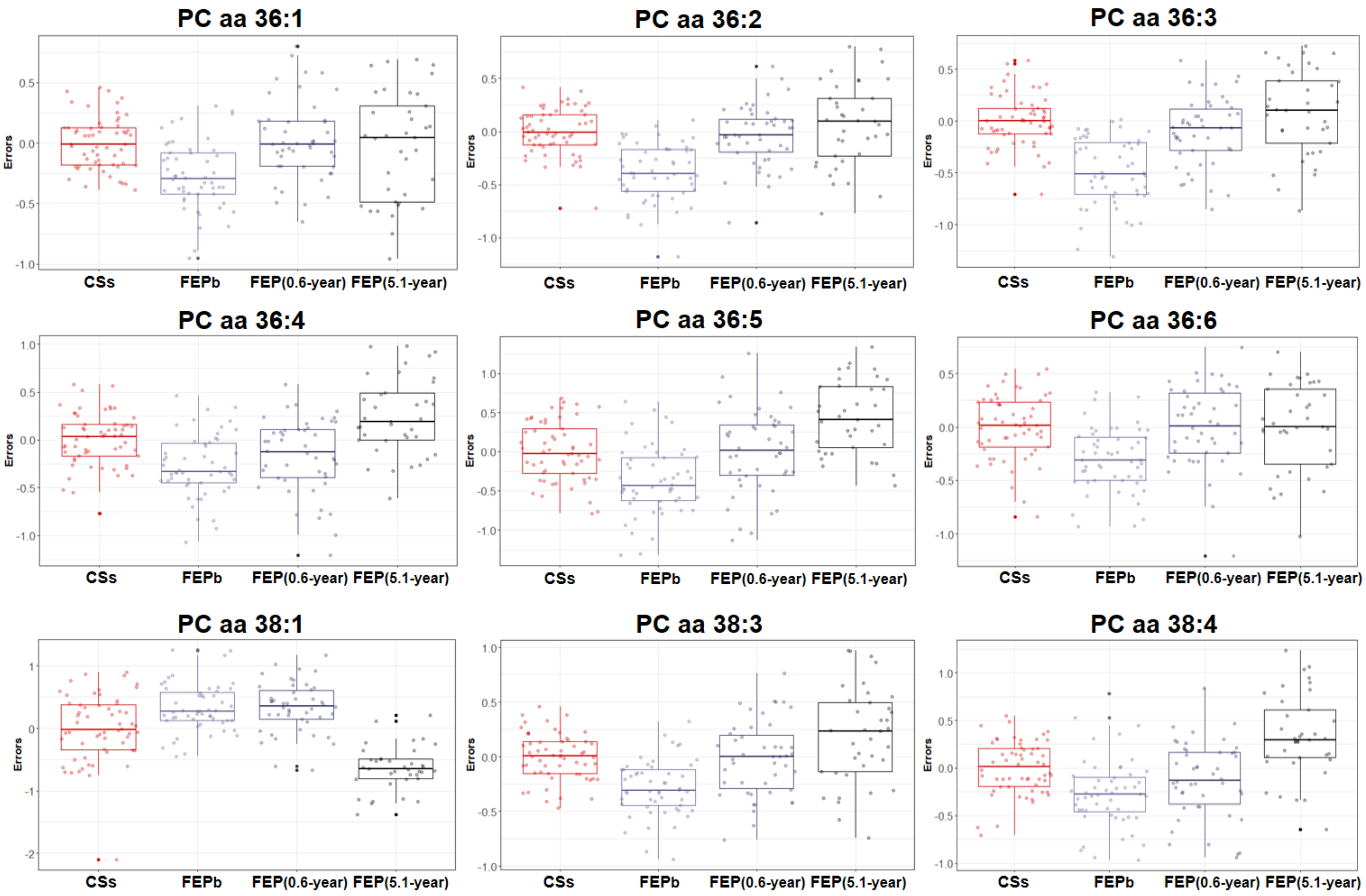
PC aa 36:1
PC aa 36:2
PC aa 36:3
PC aa 36:4
PC aa 36:5
PC aa 36:6
PC aa 38:1
PC aa 38:3
PC aa 38:4
Errors
CSs
FEPb
FEP(0.6-year)
FEP(5.1-year)

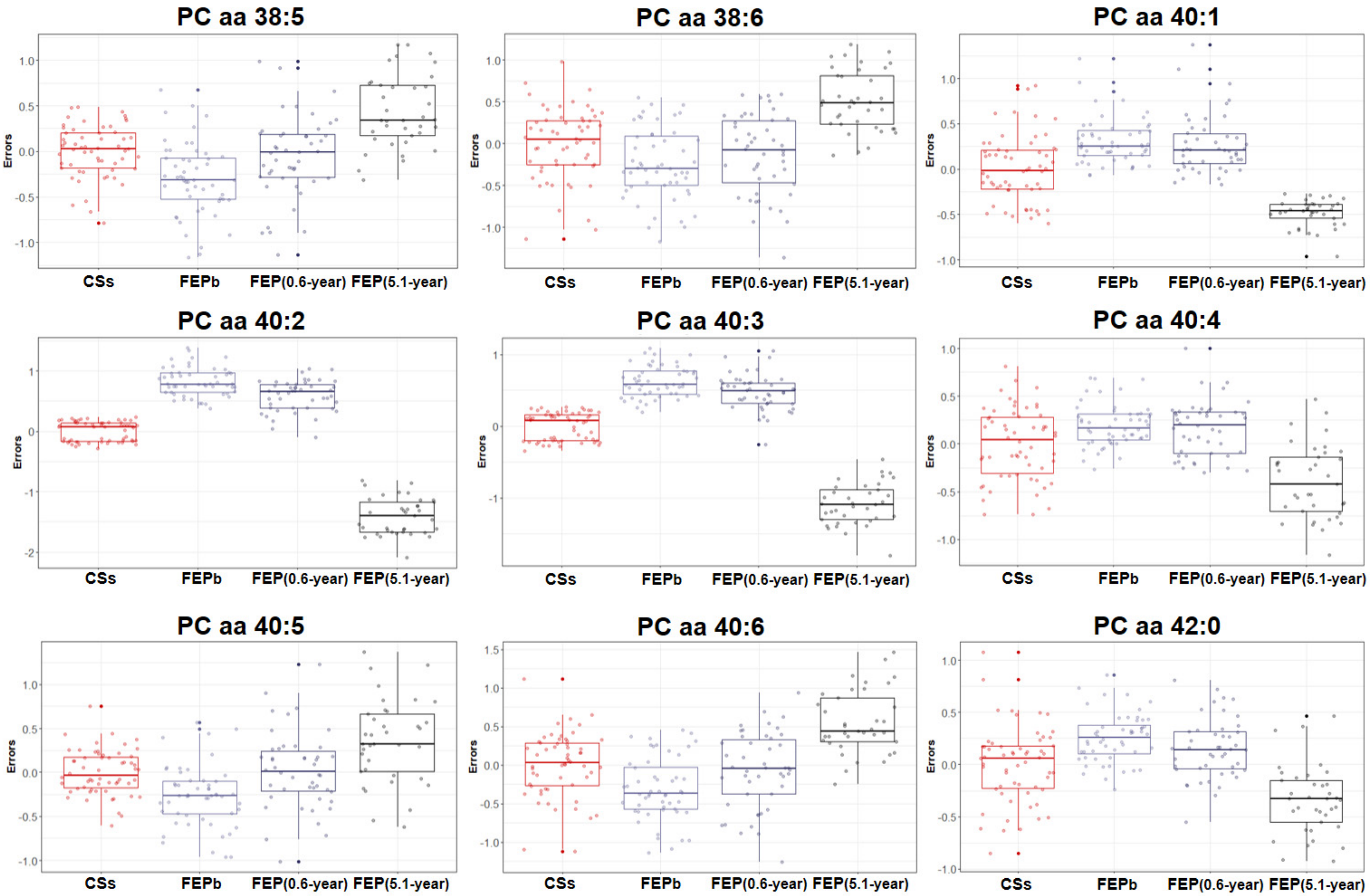
PC aa 38:5
PC aa 38:6
PC aa 40:1
PC aa 40:2
PC aa 40:3
PC aa 40:4
PC aa 40:5
PC aa 40:6
PC aa 42:0
Errors
CSs
FEPb
FEP(0.6-year)
FEP(5.1-year)

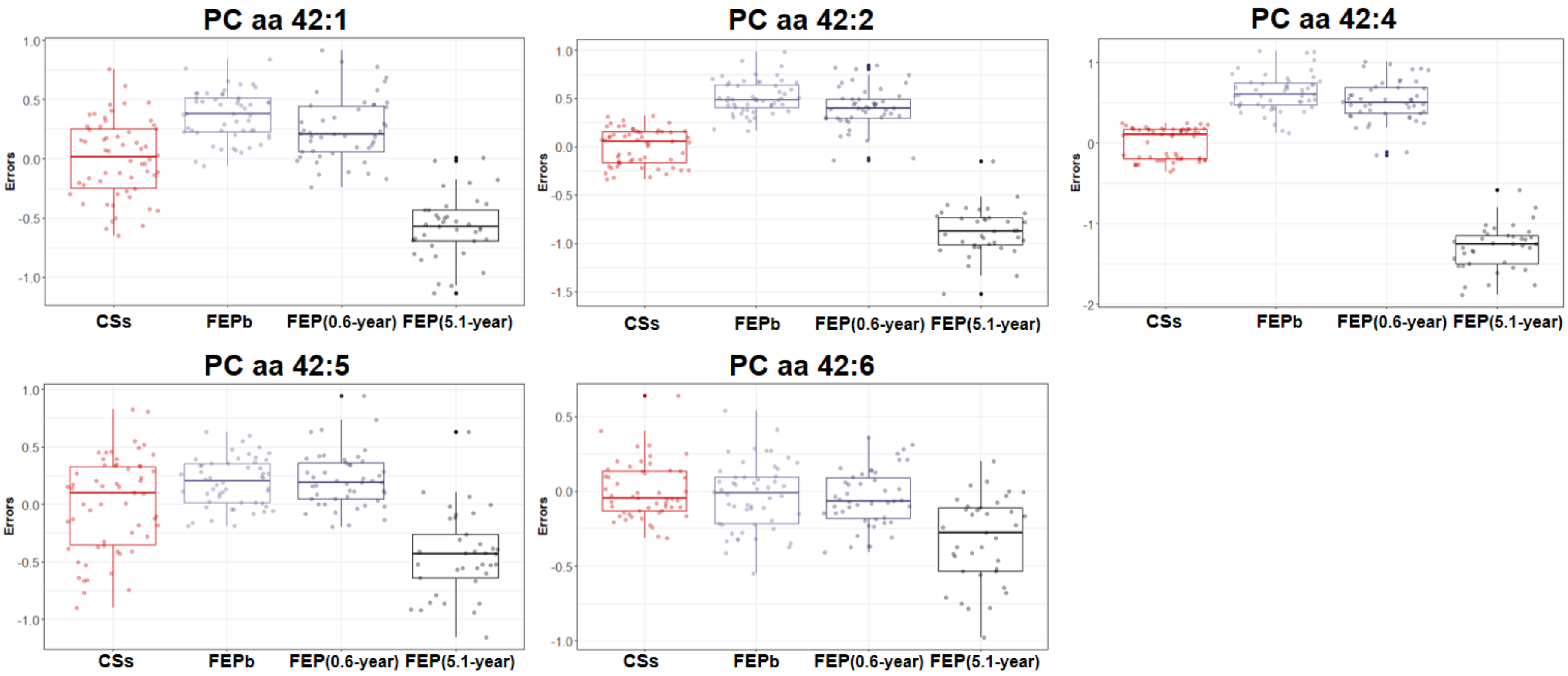

Figure S1. Boxplots of the variation of the prediction errors of log-transformed levels of eCBs, eCB-like compounds, their ratios and BMI (derived by regressing out covariate effects) for control subjects (CSs) and first episode (FEP) patients at baseline (FEP$_b$, before treatment with antipsychotics (AP)), after 0.6-year (FEP$_{(0.6\text{-year})}$), and after 5.1-year (FEP$_{(5.1\text{-year})}$) treatment with AP. The solid horizontal line in each box represents the median. The area above and below the line represents the 50th to the 75th and the 25th to the 50th percentiles, respectively. The whiskers extend to the highest and lowest values contained within 1.5 times the interquartile range of the data. Each calculated error is represented as a dot.
